# Genetics implicates overactive osteogenesis in the development of diffuse idiopathic skeletal hyperostosis

**DOI:** 10.1038/s41467-023-38279-x

**Published:** 2023-05-08

**Authors:** Anurag Sethi, J. Graham Ruby, Matthew A. Veras, Natalie Telis, Eugene Melamud

**Affiliations:** Calico Life Sciences, LLC, South San Francisco, CA 94080 USA

**Keywords:** Medical genetics, Epidemiology, Genome-wide association studies

## Abstract

Diffuse idiopathic skeletal hyperostosis (DISH) is a condition where adjacent vertebrae become fused through formation of osteophytes. The genetic and epidemiological etiology of this condition is not well understood. Here, we implemented a machine learning algorithm to assess the prevalence and severity of the pathology in ~40,000 lateral DXA scans in the UK Biobank Imaging cohort. We find that DISH is highly prevalent, above the age of 45, ~20% of men and ~8% of women having multiple osteophytes. Surprisingly, we find strong phenotypic and genetic association of DISH with increased bone mineral density and content throughout the entire skeletal system. Genetic association analysis identified ten loci associated with DISH, including multiple genes involved in bone remodeling (*RUNX2, IL11, GDF5, CCDC91, NOG, and ROR2*). Overall, this study describes genetics of DISH and implicates the role of overactive osteogenesis as a key driver of the pathology.

## Abstract (web summary)

The genetic contribution to risk of Diffuse Idiopathic Skeletal Hyperostosis has been unclear. Here, the authors find genetic variation associated with Diffuse Idiopathic Skeletal Hyperostosis, finding phenotypic and genetic association with increased bone mass throughout the skeletal system.

## Introduction

Musculoskeletal conditions are the largest contributor to years lived with disability worldwide, often due to reduced mobility with age. Within musculoskeletal conditions, DISH is the second most common form of arthritis after osteoarthritis occurring in ~23% of men and ~13% of women over age 50^1^. It is characterized as a non-inflammatory spondyloarthropathy manifesting in ectopic calcification of spinal tissues. Unlike many types of arthritis, DISH occurs more often in men than women, affecting about 1.5× the number of men than women^2^. Yet, despite its high prevalence, health professionals are often unfamiliar with DISH, and it is underdiagnosed and often misdiagnosed^3^.

The disease is considered to be idiopathic with unknown etiology^4^. As DISH is often found to be comorbid with metabolic disorders, it is possible that DISH is at least partly driven by a shared biochemical pathway with metabolic disorders such as hyperlipidemia and hyperglycemia. Various forms of osteoarthritis are also found in DISH patients, and it is possible that the disease is related to bone restructuring and cartilage loss. Drawing an analogy to Ankylosing Spondylitis (AS)—an inflammatory spine calcification condition, it is possible that inflammation may play a role in DISH^5^. Currently, there are no disease-modifying treatments available to patients, and can only be treated through surgical resection of osteophytes.

In the clinic, DISH is most commonly observed as an incidental finding in radiological scans in non-symptomatic individuals. The radiological diagnosis relies heavily on the severity of ossification, and a number of diagnostic criteria have been established to determine if the severity is sufficiently high to constitute a unique diagnosis. The most commonly used diagnostic criteria for DISH was first developed by Resnick and Niwayama in 1976^6^. It defines DISH as flowing calcifications and/or ossifications along the anterolateral aspect of four contiguous vertebral segments, without loss of IVD height in the absence of bony ankylosis of facet joints (to specifically differentiate from ankylosing spondylitis). However, it is increasingly recognized that the ossification observed in DISH progresses continuously but slowly over time leading to the development of continuous quantitative measures of DISH severity^7,8^.

Symptomatic DISH often manifests in back pain and stiffness. The pathology is diagnosed fairly late, at the point when physical impairment is so severe as to limit the motion of the thoracic spine^9,10^. In advanced cases, other presentations include thoracic spine pain, dysphagia, and compression of the spinal cord and nerve roots^11,12^. DISH is also associated with an increased risk of spinal fractures^13^ and postsurgical heterotopic ossifications^3^. Furthermore, DISH patients have an increased history of upper extremity pain, and more extremity and spinal stiffness compared to healthy control patients. Specifically, both bending ability and grip strength are reduced in DISH patients compared to other back pain patients^10^.

There have been no prior GWAS studies for DISH, and to what extent genetics contributes to the development of DISH is not known. A single nucleotide polymorphism in the COL6A1 gene has been correlated to the presentation of DISH, although only in a Japanese sub-population which also has a strong prevalence of the related spine calcification disorder OPLL^14^, making it difficult to ascertain the relative contribution of this polymorphism to each disorder. Epidemiologically, it is commonly comorbid with numerous disorders including metabolic syndrome, cardiovascular disease, diabetes mellitus, and hypertension^15^, but to what extent the genetic risks are shared is also unknown.

The availability of large-scale DXA imaging in the UK Biobank with corresponding genetics and biochemistry data for all individuals provides a unique opportunity to uncover risk factors and genetic drivers of the disease. As DISH is severely underdiagnosed, we first trained a machine learning model to automatically detect osteophytes in lateral DXA scans. We then scored the severity of pathology and carried out both phenotypic and genetic characterization of pathology across ~40,000 participants. We show that pathology is severely underdiagnosed and highly prevalent. We also find that increased bone mineral density across the whole skeletal system was among the strongest predictors of DISH and that several musculoskeletal traits share a common genetic architecture with DISH. We further carry out GWAS, fine map and functionally annotate potential loci involved in the formation of DISH. Overall, our analysis puts forward a hypothesis that overactive osteogenesis plays a key role during the development of DISH. We further support this hypothesis using Mendelian Randomization (MR) technique to test for a causal association between gene expression and the development of DISH (outcome).

## Results

### Predicting prevalence and severity of DISH using machine learning

In our analysis we quantify the extent of osteophyte bridge formation across all visible vertebrae in the ~40,000 UK Biobank lateral-view DXA scans using a continuous measure of DISH severity (flow score) developed by Kuperus et al.^7^. The baseline characteristics of the cohort are listed in Table 1. DISH flow scores are useful for detecting DISH at the early stages of development as well as for early diagnosis of DISH^16^. To detect osteophyte formation in large imaging datasets, we automated the manual annotation of the DISH flow score by developing an object-detection and multi-category classification machine learning (ML) pipeline.

**Table 1:** Baseline characteristics of imaging subcohort

	UK biobank cohort baseline visit (2008–2010)	Imaging subcohort baseline visit (2008–2010)	Imaging subcohort first imaging visit (2014+)
*n*	502,604	40,346	40,346
Age (years)	56.3 (8.1)	55.0 (7.5)	63.7 (7.6)
% Females	54.40%	51.70%	51.70%
BMI	27.43 (4.80)	26.54 (4.40)	26.61 (4.80)
Smoker (current)	10.50%	6.36%	3.55%
Smoker (previous)	34.50%	32.96%	33.84%
*Comorbidities*			
% Hypertension	7.88%	6.44%	16.83%
% T1D	0.42%	0.26%	0.38%
% T2D	1.83%	1.57%	3.86%
% CKD	0.13%	0.71%	1.96%
% Dorsalgia	15.56%	10.31%	15.01%
% Scoliosis	0.81%	0.32%	0.49%
% Ankylosing Spondylitis	0.41%	0.21%	0.32%
% Kyphosis	0.19%	0.02%	0.05%
% Spondylosis	0.07%	0.03%	0.04%
% Knee Osteoarthritis	8.72%	1.38%	3.96%
% Hip Osteoarthritis	5.07%	0.20%	0.62%
% DISH EHR	0.0085%	0.0024%	0.0024%
*Biomarkers*			
Glucose (mmol/L)	5.12 (1.24)	4.99 (1.24)	
HbA1c (mmol/mol)	35.2 (6.78)	35.0 (6.78)	
Trig (mmol/L)	1.75 (1.45)	1.65 (1.03)	
LDL (mmol/L)	3.56 (0.87)	3.58 (0.87)	
HDL (mmol/L)	1.45 (0.38)	1.47 (0.38)	
Cholesterol (mmol/L)	5.69 (1.14)	5.72 (1.14)	
Serum Phosphate (mmol/L)	1.16 (0.16)	1.16 (0.16)	
Serum Calcium (mmol/L)	2.38 (0.09)	2.38 (0.09)	
Serum Creatinine (umol/L)	72.31 (18.55)	72.34(18.55)	
Cystatin C (mg/L)	0.91 (0.18)	0.87 (0.12)	
VitD3 (nmol/L)	48.61 (21.11)	49.73 (21.11)	

Baseline characteristics of the UK Biobank cohort and the imaging subcohort. The following second level ICD10 codes from the electronic health records of all participants were used to identify the number of participants with different comorbidities prior to the appropriate visit to the center: I10 - Hypertension, E10 - Type 1 Diabetes (T1D), E11 - Type 2 Diabetes (T2D), N18 - Chronic Kidney Disease (CKD), M54 - Dorsalgia, M41 - Scoliosis, M45 - Ankylosing Spondylitis, M40 - Kyphosis, M47 - Spondylosis, M17 - osteoarthritis of knee, and M16 - osteoarthritis of hip. *BMI* body mass index, HbA1c - glycated hemoglobin, *Trig* triglycerides, *LDL* low density lipoprotein, *HDL* high density lipoprotein. The imaging cohort revisited the centers for imaging ~8 years after the baseline visit for all UK Biobank cohort participants.

**Fig. 1:**
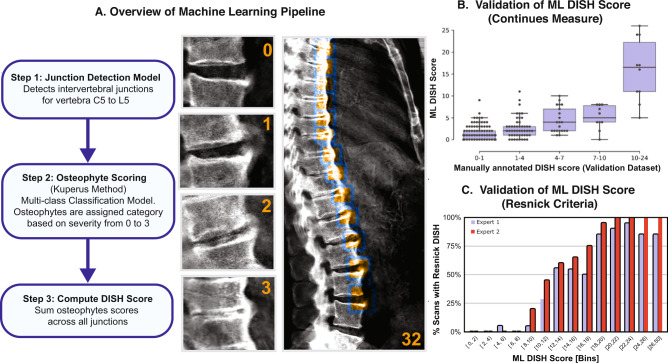
Overview of the machine learning results and validation. **A** Representative images of individual osteophyte flow scores and calculation of a total DISH score across all vertebrae. **B** Comparison of machine learning derived DISH scores with median scores from three annotators in the validation dataset (*n* = 300 images). The centerline of the box represents the median ML DISH score while the whiskers indicate the 25th and 75th percentile of the ML DISH score within that category. Individual comparison between annotators and ML scores can be found in Supplementary Fig. 3. **C** Comparison of ML scores with assessment by two expert radiologists based on Resnick diagnostic criteria for DISH. Majority of individuals above a score of 12 have DISH based on the Resnick criteria.

In the first step, the ML model detects the anterior side of each intervertebral disc space. The fourteen highest-confidence regions between C1 and L4 were used to define sub-images that were individually fed to an image-classification ML model, with categories corresponding to flow scores of zero (absence) to three (completely fused), see Fig. 1A. The scores corresponding to those categories were summed across all the vertebrae in each spine to produce an aggregate score per participant—the DISH Flow score. Representative images with corresponding ML scores are shown in Supplementary Fig. 4.

To create an independent validation dataset to evaluate the performance of the ML model, three annotators manually inspected 300 DXA scans at each intervertebral disc region and carried out aggregate flow score calculation. There was strong inter-curator agreement (Correlation > 0.8). The machine learning pipeline performed well (Fig. 1B), matching the accuracy of human annotators (Correlation > 0.8, *p* value = 8.5e−42, Supplementary Fig. 3). A detailed description of ML Methods can be found in the Supplementary Note 1 and code repository github.com/calico/DISH.

A number of related skeletal disorders are also known to form osteophytes and fusions between vertebrae (such as Ankylosing spondylitis and Spondylosis). These conditions are specifically differentiated from DISH based on the criteria that osteophytes in DISH are not accompanied by a reduction of intervertebral disc spacing and a threshold of three complete osteophyte fusion events of three adjacent vertebrae^6^. DISH flow scores are a more continuous measure of DISH severity and do not place such restrictions on the scoring scheme. Consequently, we assessed how our ML-generated DISH flow scores correspond to the DISH diagnostic events as judged by two independent expert radiologists.

To carry out this analysis, we randomly selected 20 representative images from different ML score bins representing varying levels of DISH severity. Experts were blinded to the ML scores, and were asked to evaluate the presence/absence of DISH based on Resnick criteria. If loss of vertebral spacing was observed, radiologists were asked to further evaluate if osteophyte formation might be due to related musculoskeletal disorders (Supplementary Data 1). The results of the evaluation are shown in Fig. 1C. There is a strong sigmoid curve representing the relationship between ML scores and DISH diagnoses by experts. Approximately 50% of individuals with a score of ~12 would qualify as having DISH by Resnick criteria, and more than 90% would qualify above a score of 20.

### DISH is severely underdiagnosed pathology

**Fig. 2:**
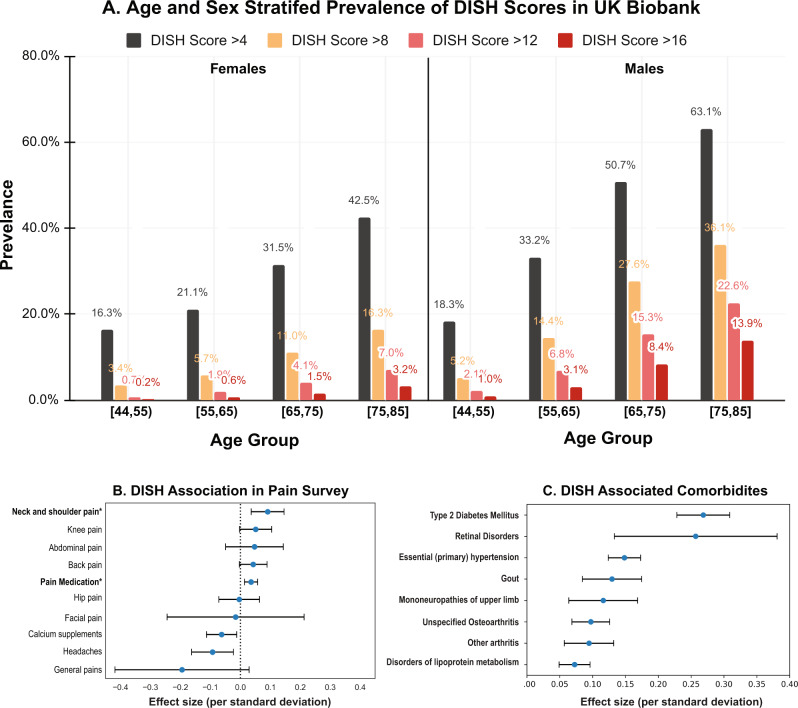
DISH Prevalence. **A** The distribution of DISH scores in the UK Biobank Imaging cohort (~40 K participants) stratified by age and gender. Men are more likely to present with multiple osteophytes than women. There is a strong age dependent increase in DISH scores. **B** Association of DISH with self reported pain questionnaires in multivariate adjusted (age, sex, age*sex, age^2, township deprivation index, smoking, ethnicity) logistic model. DISH is linked to increased use of pain medications and Neck and shoulder pain. The number of participants who replied to these questions in the questionnaire during the imaging visit varied widely from 2360 for stomach pains to 41,233 participants for taking pain medication and the logistic regression is performed with information from all participants who answered the questionnaire. **C** Association of DISH with pre-existing comorbidities in EHR records in multivariate logistic model (same adjustments as above). DISH is significantly linked to metabolic diseases, renal disorders and other forms of arthritis (*n* = 41,233). The blue dots represent the mean effect size, the error bars represent 95% confidence intervals.

Based on these results, we applied an ML scoring algorithm to ~40,000 DXA scans in the UK Biobank acquired at the first imaging visit (2014+), and were able to estimate the prevalence of pathology in the cohort (Fig. 2A). We estimate that ~12% of the population between the ages of 45–85 has multiple fusion events (flow score above 1 std. dev ~8, Supplementary Fig. 5A). There is a strong nonlinear age and sex-dependent increase in the pathology (Supplementary Fig. 5B). Above the age of 45, ~20% of men had scores >8, and ~8% of women had scores >8. A full breakdown of age by sex prevalence can be found in Supplementary Data 2.

Based on these statistics we estimate that ~65,000 participants (~18,000 women,~47,000 men) in UK Biobank have a moderate form of DISH, and ~34,900 participants (~9100 women, ~24,800 men) would meet Resnick criteria (see Methods). Yet, DISH remains significantly underdiagnosed, with only 43 EHR diagnostic records in the whole UK Biobank (ICD10 M4810-M4820, Supplementary Data 3). None of the participants in the imaging cohort were diagnosed with DISH.

### Severity of DISH is associated with pain and osteoarthritis incidence

DISH patients are thought to suffer from joint pain, reduced flexibility, and reduced lung function^17^ which are also commonly experienced by the elderly with other forms of arthritis. Given that DISH is severely underdiagnosed, it is important to evaluate the association of DISH with these symptoms and other comorbidities to clarify which symptoms are reflective of radiologically-defined DISH.

We used data from pain questionnaires and diagnosed pre-existing conditions during the imaging visit to assess the contribution of DISH to these pathologies (Supplementary Fig. 8). We find that DISH is a strong predictor of pain—people with higher DISH flow scores are more likely to experience neck pain (OR = 1.1 *p* value < 0.00114), and have increased regular usage of non-steroidal anti-inflammatory drugs (NSAIDs) (OR = 1.05, *p* value < 0.00156). However, since the use of pain medications can be attributed to multiple underlying conditions, whether this is specifically due to DISH or other comorbidities that coexist with DISH is hard to determine.

More broadly we find that DISH is comorbid with a broader set of metabolic and musculoskeletal disorders. People with higher DISH flow scores are more likely to be diagnosed with diabetes (OR ~ 1.3), obesity (OR ~ 1.1), mononeuropathies of the upper limb (OR ~ 1.1), and other forms of osteoarthritis (OR ~ 1.1) (Supplementary Fig. 9). Accordingly, individuals with DISH are more likely to have elevated levels of Urate, C-reactive protein, Hemoglobin A1C, and triglycerides (Supplementary Fig. 10). It has been previously shown that DISH is linked to metabolic disorders, but the mechanism of association remains unknown^18^.

### Higher bone mineral content as a risk factor for DISH

**Fig. 3:**
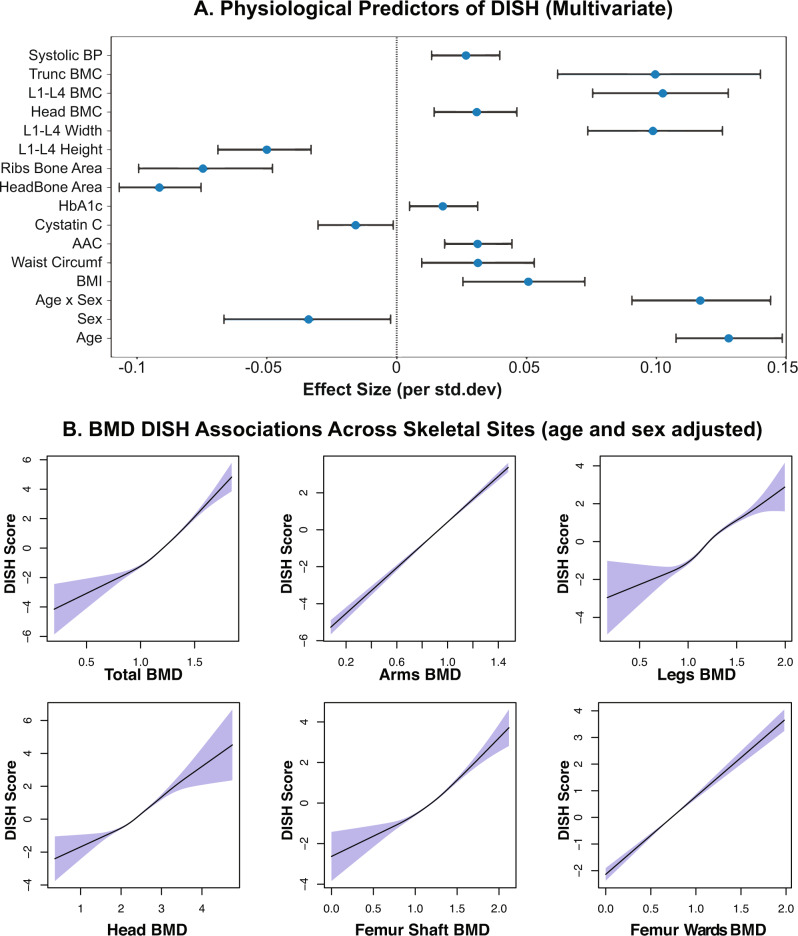
DISH Bone Density Associations. **A** Multivariate LASSO regression identifies independent physiological predictors of DISH (*n* = 41,233). Only features where 95% credible intervals do not overlap zero effect size are shown. Among the strongest risk factors are age, sex, and various BMC measures. The blue dots represent the mean effect size, the error bars represent 95% confidence intervals in the regression model. **B** Increased bone mineral density (BMD) across the entire skeletal system is associated with increased DISH score. Plot shows age and sex adjusted spline fits (GAM model) across multiple distal sites. Association of DISH with BMD in the Head, Femur Shaft, and Femur Ward’s skeletal sites (areas not known to form osteophytes) suggest that BMD is an independent risk factor for osteophyte formation. The shaded regions represent the 95% confidence interval of the spline fit.

To gain a better understanding of independent risk factors that are predictive of DISH, we constructed a univariate (see Supplementary Note 2) and multivariate linear models of the DISH flow score using baseline characteristics of the imaging cohort. We initially used a Bayesian least absolute shrinkage and selection operator (LASSO) algorithm (see Methods) to identify a set of risk factors that predict DISH conditional on each other. DISH flow score was predicted using a combination of metabolic, inflammatory, and musculoskeletal risk factors (Fig. 3A). Among the most prominent risk factors are age and male sex, as well as a number of biomarkers of metabolomics disorders such as systolic blood pressure (SBP), HbA1C, BMI, as well as multiple bone traits such as trunk BMC, L1-L4 BMC, and head BMC.

Independent associations of DISH with age and metabolic disorders have been previously reported^10,19^, but we were surprised to find that DISH is associated with an increase in BMC. The effect of increased BMC on DISH is similar to the effect of age on DISH (${\beta }_{{BMC}}$ ~ 0.1 vs ${\beta }_{{Age}}$ ~ 0.12). We repeated the correlation analysis for bone measurements across the entire skeletal system (Supplementary Figs. 6 and 7) and found strong associations of DXA-based BMD and BMC measures with skeletal sites throughout the body.

Nevertheless, to investigate further if BMD association might be due to unaccounted osteophyte contribution, we checked to see if BMD association persists in sites that do not form osteophytes such as the head and femur shaft. As can be seen in Fig. 3, the association with BMD remains robust even in sites that do not form osteophytes. Overall, this observation strongly suggests that the shared etiology of BMD and DISH pathology is unlikely to be an artifact of measurements but rather a biologically driven phenomenon. To get a better understanding of whether these phenotypic correlations can be explained by shared genetics we performed genome-wide association analysis (GWAS), genetic correlation, and Mendelian randomization analysis described in the next section.

### Genome-wide associations point to overactive osteogenesis as a key risk factor for the development of DISH

**Fig. 4:**
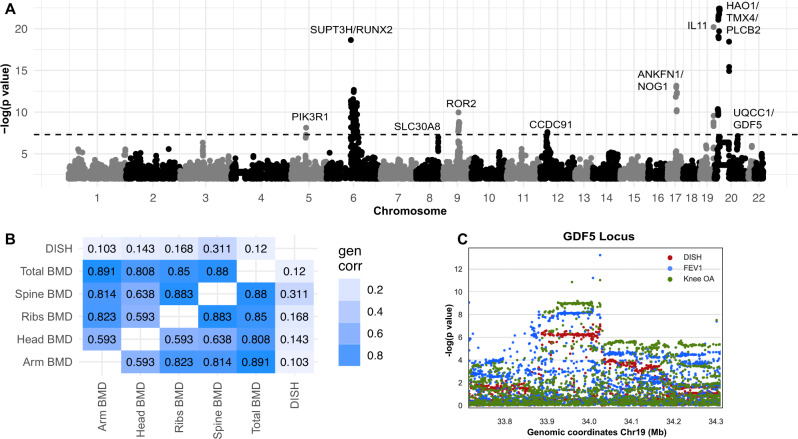
Genetic associations and genetic correlation. **A** Manhattan Plot of DISH GWAS. Associations reaching genome-wide significance below *p* value < 5e^−8^ were labeled. The *p* value is the two-tailed test for association of trait with variant. **B** Genetic correlation between DISH and other phenotypes derived from DXA scans, estimated using LD score regression from UK Biobank summary statistics. **C** GDF5 locus showing a clear overlap of genome-wide significant signals with DISH, Osteoarthritis (OA), and lung Forced Expiratory Volume in one second (FEV1). The *p* value is the two-tailed test for association of trait with variant.

To identify common genetic variants that contribute to the risk of developing DISH, we performed a GWAS of the DISH flow scores using a linear mixed effect model implemented in the BOLT-LMM package. GWAS was limited to European ancestry and adjusted for age, age^2^, sex, and age × sex, as well as ancestral relatedness following best practices as described by authors^20^. The plot of significant associations is shown in Fig. 4A. Estimated SNP-based heritability is 21.6% (s.e. 1.8%). We considered a possibility that sex-specific differences in DISH could be falsely inducing genetic associations^21^, and carried sex-stratified analysis of both GWAS and genetic correlations (Supplementary Data 4 and Supplementary Fig. 12). Despite the reduction in power, we observed consistency of genetic associations in both sexes. To establish the shared genetics between DISH and BMD/BMC, we also computed GWAS for 66 bone traits quantified using DXA scans (see Methods). The heritability of bone traits ranged from 0.2 to 0.4 (Supplementary Data 5) which is comparable to heritability of DISH and its comorbidities (Supplementary Data 6).

As both DISH and bone traits show moderate heritability, we then considered the question to what extent these traits are genetically correlated. Using LD score regression (Bulik-Sullivan, Loh, Finucane et al., 2015), we found that DISH was strongly genetically correlated with numerous BMD measures, including spine BMD (LD-score correlation 0.286), and spine BMC (LD Score correlation 0.378). Most importantly, DISH was also significantly genetically correlated with BMD measurements in distal parts of the skeleton including ribs BMD (~0.17), skull BMD(~0.14), and arms BMD (~0.10) (Fig. 4B). The strong genetic correlation between BMD and DISH supports a hypothesis that these traits share genetic architecture.

To get a better understanding of molecular mechanisms that may underlie genetic associations, we carried out GWAS-based gene set enrichment using three different algorithms LDSC^22^, GARFIELD^23^, and DEPICT^24^. Here we did not find significant enrichment of musculoskeletal phenotypes largely due to the underrepresentation of musculoskeletal expression and chromatin mark data in public datasets used by these tools. We carried out manual annotation of genes in proximity to GWAS signals using published literature. Summary of our findings is reported below and Table 2, and PheWAS Supplementary Figs. 14 and 15.

**Table 2:** DISH GWAS associations

Implicated loci	Prior GWAS associations
RUNX2/SUPT3H	*SUPT3H gene overlaps with the master transcription factor involved in osteogenesis RUNX2.* Associated with OPLL, eBMD, height, hip OA, hip replacement, intertrochanteric-shaft area, serum urate, eGFR, male pattern baldness, and BMI.
CCDC91	*CCDC91 is a known target of the transcription factor RUNX2*^35^. Associated with OPLL, height, eBMD, lumbar spine bone area, neck bone area, lung function, COPD, bone morphology, fat free mass measures, BMD, and breast cancer.
UQCC1/GDF5	*GDF5 is a bone morphogenetic protein involved in joint and bone repair and development (Storm and Kingsley 1999).* Associated with height, cholesterol, triglycerides, eGFR, eBMD, spine and trochanter area, fat free mass, osteoarthritis, knee replacement, coronary artery disease, stroke volume, arthropathies, brain region volumes, SBP, prostate cancer, and hand grip strength.
ANKFN1/NOG	*Noggin is a glycoprotein that binds and antagonizes BMPs*^29^. Associated with fat free mass measures, eBMD, bone deformities, lung function, chronotype, and eGFR.
POLD3/CHRDL2	*CHRDL2 inhibits BMP signaling and bone formation (Nakayama* et al. *2004).* Associated with height, bone area, diastolic blood pressure, hand grip strength, eGFR, knee replacement, knee OA, and fat free mass measures.
IL11	*IL11 has a dual role in osteogenesis. It is involved in determining survival of pre-osteoclast cells and is a part of mechanical sensing of bone formation.* (Hill et al. 1998). Associated with height, hip replacement, hand grip strength, HbA1c, cholesterol, lung function, osteoarthritis, and fat free mass.
HAO1/TMX4/PLCB1	Associated with OPLL, Hand OA, Lumbar Spine Area, Heel BMD, and eGFR.
ROR2	*Ror2 tyrosine kinase receptor homodimerizes and promotes osteoblast differentiation and bone formation*^64^. Associated with calcium, chronotype, height, systolic blood pressure, and facial morphology.
SLC30A8	*SLC30A8 is largely an endocrine pancreas-restricted zinc transporter previously linked to T2D*^65^. Associated with T2D, HbA1c, glucose, BMI, proinsulin, triglycerides, late diabetic kidney disease, and macroalbuminuria.
PIK3R1	*PIK3R1 is involved in osteoblast differentiation through the phosphoinositide signaling cascade (McGonnell* et al. *2012)*. Associated with height, triglycerides, HDL cholesterol, eGFR, BMI, fat free mass measures, lung function, and CVD.

The list of associations for each loci based on the EBI GWAS catalog^66^, the GWAS atlas^67^, and the musculoskeletal knowledge portal^68^.

*eBMD* estimated BMD, *OA* osteoarthritis, *T2D* Type 2 Diabetes, *eGFR* estimated glomerular filtration rate, *COPD* Chronic Obstructive Pulmonary Disease, *BMI* body mass index, *SBP* systolic blood pressure.

### Fine mapping and colocalization of GWAS hits

To gain a better understanding of the common molecular mechanisms that underlie the genetic risk of DISH and related musculoskeletal phenotypes, we carried out fine mapping and colocalization analysis.

**Fig. 5:**
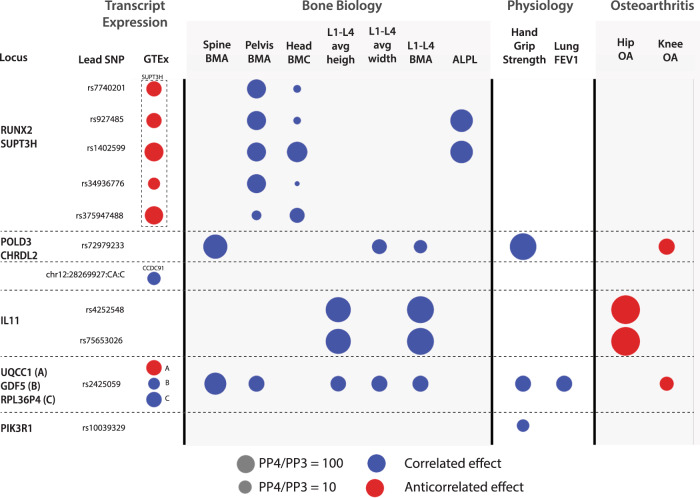
Genetic Colocalization Analysis. The colocalization of genetic signals of DISH with different related traits as well as gene expression of neighboring genes from GTEx in any tissue. A colocalized signal for a particular trait is represented by a circle on the corresponding locus. The size of the circle represents the strength of the colocalization signal. PP4 is the posterior probability that both traits are caused by the same variant within this locus, while PP3 represents the posterior probability that both traits are caused by different variants within the same locus. The color of the circle represents whether the risk allele changes DISH as well as the corresponding trait in the same direction (correlated/blue) or opposite directions (anti-correlated/red). L1-L4—lumbar vertebral spine, BMA—DXA based bone mineral area, BMC—DXA based bone mineral content, FEV1—lung Forced Expiratory Volume in one second. ALPL—Alkaline phosphatase.

First, we find independent signals associated with DISH across the whole genome and then identify potential causal variants within each associated locus using a fine-mapping approach (See Methods). Here we found 19 independent risk signals containing 340 potential causal variants (posterior inclusion probability > 1%) within the ten loci below the genome-wide significance threshold of 5 × 10^−8^ (Fig. 5). Three of these ten loci (*RUNX2*, *CCDC91*, *HAO1/TMX4/PLCB1*, Table 2) have been previously associated with the related ossification pathology of the posterior longitudinal ligament (OPLL)^25^. OPLL is more prevalent in Eastern populations and often comorbid with DISH leading to the hypothesis that DISH and OPLL share a common genetic etiology^26^. One of the SNPs was localized to the coding region of *IL11*, a gene previously implicated in the development of osteoarthritis. We also observed that a number of non-coding lead SNPs were found in the proximity of *RUNX2, GDF5, CHRDL2, ROR2, PIK3R1*, and *NOG* - genes previously associated with height, bone homeostasis, and osteoarthritis^27–30^ (Table 2). Together, our genetic results implicate a number of genes associated with musculoskeletal traits to also be associated with DISH.

Second, we performed colocalization analysis with UK Biobank phenotypes and GTEx tissue eQTL to identify traits that likely share the same underlying causal variants. A summary of colocalization results is shown in Fig. 5. In GTEx analysis, 7 of the 19 lead SNPs colocalize with an expression of (*RUNX2/SUPT3H, POLD3/CHRDL2, IL11, UQCC1/GDF5, PIK3R1*), suggesting that expression of these genes might influence the development of DISH. A critical limitation of GTEx eQTL colocalization analysis for musculoskeletal traits is that GTEx does not contain eQTL for bone measurements, and observed signals must be originating from other tissues. In the UK Biobank colocalization analysis, we observed the colocalization of multiple DISH fine mapped SNPs with the genetics of bone traits and osteoarthritis. The overall direction of genetic colocalization and phenotypic correlations are consistent, with exception of IL11 and *POLD3/CHRL2* variants, where colocalization is anti-correlated with osteoarthritis, while phenotypically we see a positive correlation between DISH and OA. Below we describe colocalization results for each locus in more detail.

The first locus on chromosome 6 had eight independent signals. Five of these signals colocalize with the histone modification enzyme *SUPT3H* and one of the variants overlaps with the master transcription factor involved in osteogenesis *RUNX2* (Fig. 5). *SUPT3H* is ubiquitously expressed in multiple tissues while *RUNX2* is upregulated during processes such as osteogenesis^31^. Given shared regulatory mechanisms between *SUPT3H* and *RUNX2*^32^, it is hard to determine which of these two genes are casual for DISH.

All eight independent signals also colocalize with genetic signals for bone mineral content (BMC) and bone area for different parts of the body (Fig. 5). In addition, two of the signals also colocalize with genetic signals associated with increased levels of alkaline phosphatase, a known biomarker of bone health. This locus is also associated with musculoskeletal traits such as estimated BMD, height, hip osteoarthritis, hip replacement, and bone area as discussed in Table 2. Overall, the signal at this locus provides evidence for genetic association between increased BMC and DISH.

The second locus on chromosome 20 colocalizes with genetic signals affecting the expression of two genes (*UQCC1 & GDF5*) and a pseudogene (*RPL36P4*). GDF5 is a pleiotropic bone morphogenetic protein involved in joint and bone repair and development^28^. Alleles that reduce *GDF5* expression were implicated in several processes (Table 2) including the development of osteoarthritis^33,34^. Our colocalization analysis shows an association between DISH with knee osteoarthritis as well as other bone-related traits. However, the genetic effects of DISH are anti-correlated to the genetic effects of osteoarthritis, showing that there is a delicate balance between overactive and reduced osteogenic activities resulting in partitioning risk between bone-related diseases.

The third locus on chromosome 12 colocalizes with genetic signals affecting the expression of *CCDC91* in multiple tissues. CCDC91 is a known target of the transcription factor RUNX2^35^ and variants affecting the expression of *CCDC91* have been implicated in musculoskeletal traits such as height, estimated BMD, spine bone area, neck bone area, OPLL^36^, osteoporosis^37^, and osteoarthritis^38^. Upregulation of *CCDC91* is thought to play a role in the progression of OPLL by inducing ossification under mechanical stress^39^.

The fourth locus on chromosome 19 occurs within the coding region of *IL11* and this signal colocalizes with several bone-related traits and is a known risk factor for developing osteoarthritis (Fig. 5). *IL11* has a dual role in osteogenesis. It is involved in determining survival of pre-osteoclast cells and is a part of the mechanical sensing of bone formation^40,41^. The rs4252548 SNP associated with DISH results in a missense coding variant R112H that reduces the thermostability of IL11 and is implicated in several musculoskeletal traits such as height and osteoarthritis^42^. We hypothesize that the consequence of the mutation is to increase osteogenesis through an unknown mechanism that eventually leads to the development of DISH.

The fifth locus on chromosome 11 is close to DNA polymerase 3 (*POLD3*) and Chordin-like protein 2 (*CHRDL2*). *CHRDL2* is expressed in chondrocytes within joint cartilage and connective tissues and is associated with multiple musculoskeletal traits such as height, bone area, and osteoarthritis (Table 2). *CHRDL2* is overexpressed in osteoarthritic joint cartilage where it is thought to play a protective role in the development of osteoarthritis^43,44^. The mechanism of this locus’s action on DISH is unknown, but *CHRDL2* binds directly to bone morphogenetic proteins (BMPs) to prevent BMP signaling and bone formation^44^. Consistent with its function in bone homeostasis, the genetic signal for DISH at this locus colocalizes with spine bone traits.

The sixth locus associated with DISH is located on chromosome 5 close to phosphoinositide-3-kinase regulatory subunit 1 (*PIK3R1)* gene. PIK3R1 is involved in osteoblast differentiation through the phosphoinositide signaling cascade^43^. The role in DISH is likely to be through increased osteogenic potential. Consistent with this hypothesis, *PIK3R1* is also associated with other musculoskeletal traits such as height (Table 2). Our analysis also shows that genetic signals for DISH and hand grip strength colocalize at this locus (Fig. 5).

The remaining four loci did not colocalize with any musculoskeletal traits or eQTL loci from GTEx. Overall, the colocalization analysis reveals that the causal variants associated with DISH are also associated with multiple musculoskeletal traits and osteoarthritis.

### Mendelian randomization supports a causal role of GWAS hits

Genetic associations of loci with DISH phenotype are not sufficient to establish the causal role of genes in developing DISH. It is possible that unaccounted confounding signals are driving the association between genotype and phenotype. To get a better estimate of the potential causal involvement of a given loci with DISH, it is possible to use the instrumental variable Mendelian Randomization model to carry out a form of mediation analysis, where genetic variants (instrumental variables) linked to gene expression (mediator) are tested for association with outcome (DISH). If a genetically linked variation in gene expression is associated with the outcome, it is possible to conclude that the gene is causally linked to the outcome (under some assumptions see Methods).

This methodology is only possible if eQTL for genes can be established. In our analysis, we carried out a two-sample Mendelian Randomization (MR) analysis using eQTL data from GTEx v7 across 44 tissues. One of the limitations of the analysis is that bone phenotypes are poorly measured in public databases, and generally bone and intervertebral expression data are not available. Thus we have to rely on eQTLs from other tissues. When multiple independent eQTLs were observed for the same gene in a single tissue within GTEx, we evaluated the causal effect of a gene on DISH using inverse variance weighted (IVW) analysis. For the genes with single independent eQTL in a tissue we used the Wald ratio test. A summary of our findings for all genes analyzed, with exception of *PIK3R1*, which didn’t have eQTL data, can be found in Table 3.

**Table 3:** Mendelian randomization analysis (Variants → eQTL → DISH)

Gene	Method	beta	*P* value
RUNX2	Wald Ratio	0.054	5.90E−04
IL11	Wald Ratio	0.18	4.33E−09
GDF5	Wald Ratio	0.059	1.15E−07
NOG	Wald Ratio	0.053	2.01E−03
ROR2	Wald Ratio	0.029	0.012
CHRDL2	Wald Ratio	0.073	0.006
CCDC91	IVW	0.14	5.54E−11
UQCC1	IVW	−0.07	4.72E−10

The *P* value is calculated using the two-tailed *t*-test and is uncorrected for multiple comparisons.

We find that most GWAS had evidence of causal involvement in the development of DISH. In the IVW analysis, expression of *CCDC91* (most significant in the heart atrial appendage tissue) and *UQCC1* (most significant in transformed fibroblast cells) were significantly associated with increased DISH. In the Wald ratio test analysis, expression of *RUNX2* (substantia niagra of the brain), *IL11* (lung), *GDF5* (basal ganglia of the brain), and *NOG* (gastroesophageal junction) play a causal role in DISH with the effect sizes shown in Table 3. We also performed a sensitivity analysis for IVW MR, and did not find evidence of horizontal pleiotropy for either *CCDC91* (*p* value = 0.4) or *UQCC1* (*p* value = 0.6). Overall, MR analysis supports the hypothesis that expression difference in six genes (out of eight tested GWAS hits analyzed) is causally linked to the development of DISH.

## Discussion

Despite the high prevalence of DISH in the elderly population, it remains a poorly characterized and underdiagnosed disease. As a consequence, the genetic and environmental risk factors that lead to the development of this pathology are not well known, and the long-term consequences of DISH pathology on health outcomes have not been systematically analyzed. There have been a number of smaller studies that looked at the prevalence of DISH^2,45,46^, but to our knowledge, this is the most comprehensive analysis of the genetic and epidemiological characterization of DISH in the general population.

Using a machine learning approach we predicted DISH flow scores for ~ 40,000 participants in UK Biobank. The algorithm automatically detects pairs of vertebral bodies and quantitates the severity of osteophyte formation between them. In our evaluation, the algorithm performed well, matching human annotators with correlation >0.8. We further showed that the DISH flow score predicted by the algorithm is capable of capturing established Resnick DISH diagnostic criteria annotated by expert radiologists.

We find that the prevalence of DISH is strongly associated with age and male gender, with approximately one in eight individuals having multiple osteophyte bridges. Despite the high prevalence, DISH is severely underdiagnosed, with only 43 recorded diagnoses in the UK Biobank EHR. Based on the results of machine learning, we estimate that around ~34,900 UKBB participants would meet the radiological Resnick criteria for DISH diagnosis.

The presence of spine osteophytes is significantly associated with neck and shoulder pain and the use of non-steroidal anti-inflammatory drugs (NSAIDs). However, as DISH is also associated with the incidence of osteoarthritis, and it is not clear if NSAID usage is due to comorbidities or DISH itself. For example, the formation of osteophytes in the spine might be correlated with the formation of osteophytes in the hips as observed in hip OA, thus increasing the usage of NSAID pain medications^47^. While further research is needed to provide a definitive answer, association with pain in the neck and shoulders in comparison to lower back pain, suggests that DISH may be contributing to the pain symptoms.

Most notably we observed a strong association of DISH with bone mineral content and bone mineral density across the entire skeletal system. We carried out multivariate analysis after identifying a set of variables that jointly predict DISH and found that association remains significant with an effect size similar to age, indicating that high BMC/BMC is an independent risk factor for DISH. This observation raises a possibility that processes that drive the formation of higher bone density, might also be driving the formation of DISH. We considered the possibility that BMD/BMC association might be a confounded presence of osteophytes in the regions of the skeleton that are known to form osteophytes. To investigate this further, we examined if association persists in regions of the skeleton that are not known to form osteophytes (skull, femur shaft), and found that association persists in these regions.

**Fig. 6:**
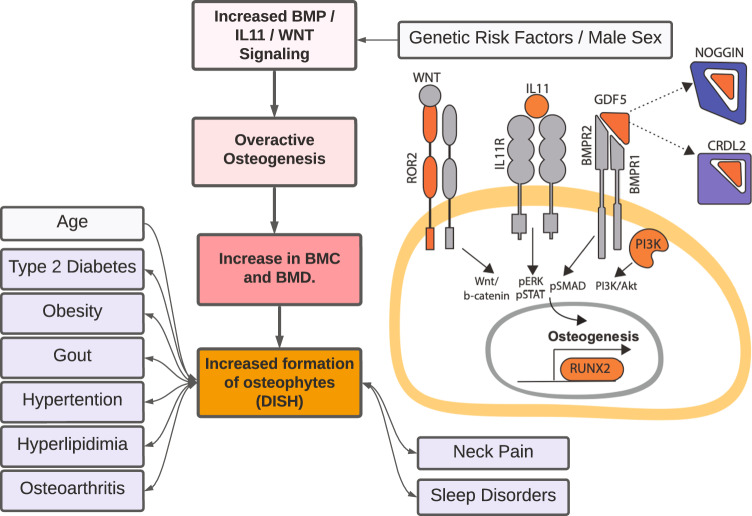
Overview of genetic and environmental risk factors associated with development of DISH. In addition to male sex and age, pre-existing conditions such T2D, obesity, and osteoarthritis are risk factors associated with DISH. Genetic analysis (GWAS, colocalization, and Mendelian randomization) points to genes involved in overactive osteogenesis as drivers of the pathology (highlighted in orange). The increase in osteogenesis, and consequence in increases in BMD and BMC measures is observed throughout the entire body. Molecular mechanisms likely involve gain of function in multiple signaling pathways such Wnt signaling, IL11 signaling, and BMP-signaling. Conversely, loss of inhibitory BMP proteins such as Noggin and CRDL2 likely increases BMP-signaling. In prognostic outcomes, DISH is associated with increased risk of metabolic and sleep disorder diagnoses.

To gain further insight into molecular processes that might play a role in the development of DISH we carried out genetic association analyses, genetic correlations, fine-mapping, and colocalization. To our knowledge, this is the first time genetics of DISH has been characterized. GWAS implicated ten novel loci, including osteogenesis master regulator *RUNX2*, BMP signaling (*CHRDL2, NOG, GDF5*), PI3K pathways (*PIK3R1*), and Wnt-signaling (*ROR2*), and *IL11* (Fig. 6). The majority of genes involved in these pathways have been experimentally shown to play a role in bone-homeostasis, supporting the hypothesis that DISH is driven by overactive osteogenesis. Using Mendelian Randomization, we further asked if a transcriptional expression of implicated genes could be causal to DISH formation, and found strong support for this hypothesis for 6 out 8 genes with eQTLs in GTEx data.

In an analysis of related conditions, we also see a significant overlap in GWAS loci (*RUNX2, CCDC91*, and *HAO1/TMX4/PLCB1*) with OPLL - a related spine ossification pathology more commonly observed in Asian populations^36^. RUNX2 haploinsufficiency is sufficient to rescue OPLL in a mouse model providing further evidence that *RUNX2* may also play a role in the development of DISH^48^. The overlap of the genetic risk factors for DISH and OPLL indicates that the two pathologies at least in part share a common genetic etiology. Of note, we did not see an overlap with genetic risk factors of Ankylosing Spondylitis, an inflammatory condition that also results in vertebral fusions, indicating that the two pathologies have distinct genetic etiology.

Overall through a combination of phenotypic and genetic associations, our study points to global overactive osteogenesis as a key mechanism for the formation of DISH. The hypothesis is supported by four lines of evidence.

First, as noted above, we see strong evidence for an association between DISH and BMD/BMC across the entire skeletal system, including sites that are not known to form osteophytes such as the femur shaft and head. The association is robust and was reproduced in sex-stratified analysis across all sites (Supplementary Fig. 12). This implies that the association of BMD/BMC with DISH is independent of osteophyte formation.

Second, we considered the possibility that the phenotypic association might be spurious as most automated BMD/BMC algorithms do not identify osteophytes and include osteophyte area in the calculation. Previous estimates suggest that the presence of osteophytes could inflate BMD in vertebral bodies by 10-12% on average^49^. The contribution of osteophytes to BMD/BMC in other skeletal regions of the body can not be completely ruled out but should be proportional to the total area of the osteophyte to the total bone area. We think that the large correlation of DISH with BMD/BMC cannot be completely explained by osteophyte formation in these sites (Fig. 3).

Third, we observe significant genetic correlations between DISH and BMD/BMC across the entire skeletal system, but not with the bone mineral area (BMA). In the spine regions, the genetic association with DISH was significant for BMD/BMC (*p* value = 5.27e−8), but not with the bone area (*p* value = 0.22). The contribution of osteophytes to the total bone area and BMC is relatively small, indicating that DISH must be occurring through a significant increase in bone content without appreciable changes to bone area. As an additional line of evidence, we asked if the genetic correlation between DISH and Spine BMD/BMC persists if we stratified cohorts into two groups with and without observed DISH. Genetic correlation between these traits remained significant in both groups with approximately the same correlation (rg = 0.27 and rg = 0.25 respectively). The only plausible explanation for the genetic correlation is that traits share a common genetic architecture beyond observed osteophyte formation.

Fourth, genetic colocalization and MR show that several key genes involved in osteogenesis such as *RUNX2* play a significant role in the development of DISH, with 9 out of 10 loci having been previously implicated in several musculoskeletal traits and core processes within bone homeostasis (Figs. 4, 5, Supplementary Figs. 14, 15, and Data 2 and 3).

### Limitations

The UK Biobank population mostly comprises Europeans, which may impact our genetic associations as well as MR results. The UK Biobank was mostly formed by volunteers and is known to have a healthy volunteer bias which might also impact the results of our analyses. Although we adjusted for age and sex (as well as their interaction), some residual participation bias might impact our results. However, this selection bias is unlikely to lead to large correlations between different bone traits and DISH.

We have tried to assess whether osteogenesis pathways are implicated in DISH using gene and tissue enrichment tools. However, due to the poor representation of bone cell types in public datasets, none of these methods showed osteogenesis as a significantly enriched pathway for either the Spine bone mineral traits or DISH (at cohort size of ~33,000).

Overall, we have carried out the largest analysis of DISH to-date and show that prevalence of DISH is substantially underappreciated in the general population. We have confirmed many of the previous epidemiological associations with pain and other comorbidities, and characterize the genetics of this previously considered idiopathic condition. Through genetic analysis, we put forward a hypothesis that overactive osteogenesis is playing a key role in the development of pathology. If this hypothesis holds, it would suggest that interventions that restore bone formation imbalance may offer a plausible path to treating this condition.

## Methods

### Imaging data

In 2006 through 2010, 503,000 adults (aged 40–70 years) were recruited from the general population in the United Kingdom into a prospective cohort study^50^. The UK Biobank aims to scan 100,000 of these participants by the end of 2023 with various imaging modalities^51^; the current analysis includes 41,233 participants for whom lumbar spine DXA imaging scans (Lunar iDXA densitometer; GE Healthcare, Chicago, Illinois) were collected for body composition and bone mineral density assessments (April 2014–September 2019). At the imaging assessment visit, information was collected on a range of demographic and lifestyle factors, including ethnicity, education, occupation, alcohol consumption, smoking status, socioeconomic status, and physical activity. Various measurements were also taken, including height, weight, waist and hip circumferences, and blood pressure. All study participants provided informed consent and the North West Multi-centre Research Ethics Committee approved the study. Further details about the procedural characteristics of the imaging data have been published online (available at http://biobank.ctsu.ox.ac.uk/crystal/crystal/docs/DXA_explan_doc.pdf).

### Machine learning

The pipeline described here scores the extent of hyperostosis in the intervertebral disks that can be observed in a lateral dual-energy X-ray absorptiometry (DXA) scan image of a human torso. As described by Kuperus et al. (2018), such hyperostosis can create bridges between vertebrae that limit flexibility and ultimately contribute to diffuse idiopathic skeletal hyperostosis (DISH).

The analysis occurs in three steps: (1) identification of anterior intervertebral junctions; (2) scoring the hyperostosis of each intervertebral junction; and (3) summing the bridge scores across the spine (Fig. 1A). Details on each of those steps are given in the Supplementary Note 1 and Supplementary Fig. 2 Source code and additional documentation are available from github.com/calico/DISH.

The first step in the analysis of DISH was the identification of the anterior portions of the intervertebral gaps along the entire spine. These are the loci where DISH-relevant bridges can form that are visible in lateral DXA images. An object-detection model was applied to this task. It was trained by transfer learning from the MobileNet ssd_mobilnet_v1 model^52^. A set of 160 images was annotated, which included 2271 boxes drawn around vertebral junctions. The annotated images were separated into training and test sets of 100 and 60 images, respectively. The performance of the object detector was evaluated in the 60-image test set using intersection-over-union (IoU) for the 14 top-scoring predicted boxes vs all of the annotated boxes, allowing each predicted box’s intersection to only be counted for its most-overlapping annotated counterpart. The average IoU across the 60 test images was 68.9% (SD 5.9%).

For each intervertebral junction, a numeric score was to be assigned according to the criteria described in Fig. 2 of Kuperus et al. ^7^. Four categories were established corresponding to the severity of hyperostosis from no visible osteophytes (category 0) to complete fusion (category 4). The image classification model was trained to classify the severity of hyperostosis across 9702 bridge images (693 DXA images) and tested on randomly selected 2800 bridge images (200 DXA scans). The training was performed using transfer learning from the efficientnet/b1 model. Cohen’s kappa value for the final model was 0.405.

The final output value of the model evaluates overall DISH-like hyperostosis across the spine. Final evaluation was performed using a hold-out set of 200 DXA images that were scored by three independent raters. Raters used the same bridge-score scheme described above. For each DXA image, those numeric scores were summed to produce the final DISH score. The evaluation was performed using the mean rater score for each DXA image. Results for the comparison between manual annotators and the ML pipeline are shown in Supplementary Fig. 3.

### Prevalence of DISH in the UKBB

The prevalence of DISH in the imaging cohort was estimated using the distribution of scores in different bins and the probability of being diagnosed with DISH by expert radiologists within each bin (Fig. 1C). The prevalence of DISH in the UKBB was naively estimated by scaling up the participants within the imaging cohort to the whole UKBB. We performed these calculations in a sex-stratified manner to estimate the number of male and female participants in UKBB that would be diagnosed with DISH according to the Resnick criteria.

### Genetics

We used the UK Biobank imputed genotypes (https://biobank.ndph.ox.ac.uk/showcase/showcase/docs/impute_ukb_v1.pdf), after excluding SNPs with minor allele frequency <1% and poor imputation quality (info value < 0.9). We removed participants who were not of European descent, exhibited sex chromosome aneuploidy, heterozygosity outliers, or genotype call rate outliers. Additionally, we removed variants with genotype missingness > 10% or that deviated meaningfully from Hardy-Weinberg equilibrium in a European ancestry cohort (HWE *p* value < 1e^−10^). In total, we considered 9,633,695 SNPs and 33,413 individuals for genetic analysis.

To conduct the genetic association study, we used BOLT-LMM^20^ using a mixed effects model with genetic relatedness derived from genotyped SNPs as a random effect to control for population structure while also adjusting for genotype SNP chip (Illumina vs Affimetrix), sex, age, age^2^, age × sex, and recruitment center as fixed effect covariates as recommended in the BOLT-LMM^53^. The GWAS was performed against standardized DISH scores per participant. We verified that the test statistics showed no inflation compared to the expectation using the genomic control lambda coefficient (1.10) and the intercept (1.004, s.d. 0.009) of linkage disequilibrium (LD) score regression (LDSC)^54^.

Volunteer-based biobanks like UKBB have sex-differential participation bias^21^. As a result, adding sex as a covariate in the GWAS might lead to collider bias that leads to significant results in GWAS on some autosomal variants that differ in frequency between the two sexes due to sex-differential participation bias. To address this issue, we have implemented a sensitivity analysis to assess the impact of sex-biased participation on our DISH and BMD GWAS. We have applied the same BOLT-LMM procedure as before but performed sex-stratified association tests using the UK Biobank data (Supplementary Data 4 and Supplementary Fig. 12). Overall, this analysis shows that adding sex as a covariate in the GWAS has minimal effects on our genetic associations with DISH and BMD.

### Genetic correlation and heritability

We estimated the heritability and genetic correlation between traits using LD score correlation (28). We accessed the repository at https://github.com/bulik/ldsc/ (version aa33296) and estimated genetic correlation and heritability using the default parameters and the –rg command and –h2 command respectively (example: ldsc.py --rg trait1.sumstats.gz,trait2.sumstats.gz --ref-ld-chr eur_w_ld_chr/ --w-ld-chr eur_w_ld_chr/ --out). We pre-harmonized the polarization of alleles to match the reference file w_hm3.snplist.

### Fine mapping

#### Identification of distinct association signals

We performed approximate conditional analysis using GCTA^54,55^, considering all variants that passed quality control measures and were within 500 kb of the locus index variant. As a reference panel for LD calculations, we used genotypes from 5000 UK Biobank participants that were randomly selected after filtering for unrelated, European participants. We excluded the major histocompatibility complex (MHC) region due to the complexity of LD structure at this locus (GRCh37::6:28,477,797-33,448,354; see https://www.ncbi.nlm.nih.gov/grc/human/regions/MHC). For each locus, we considered variants with locus-wide evidence of association (*p* value_joint_ < 10^−6^) to be conditionally independent while the genome-wide association threshold to define a locus was set at *p* value < 5 × 10^−8^.

The genomic inflation factor (λ_GC_) was 1.10 (LD score regression intercept = 1.004), indicating that there was a low possibility of false positive associations resulting from population stratification.

#### Construction of genetic credible sets

For each distinct signal, we calculated credible sets with 95% probability of containing at least one variant with a true effect size not equal to zero. We first computed the natural log approximate Bayes factor^56^, Λ_j_, for the j th variant within the fine-mapping region:

1 $${\varLambda }_{j}={{{{\mathrm{ln}}}}}\left(\sqrt{\frac{{V}_{j}}{{V}_{j+\omega }}}\right)\frac{\omega {\beta }^{2}}{2{V}_{j}({V}_{j}+\omega )}$$

where β_j_ and V_j_ denote the estimated effect size and corresponding variance. In loci with multiple distinct signals of association, results are presented using both exact conditional analysis after adjusting for all other index variants in the fine-mapping region and results from the marginal analysis that does not adjust for other index variants within the locus. In loci with a single association signal, results are presented from an unconditional meta-analysis. The parameter ω denotes the prior variance in allelic effects and is estimated as (0.15σ)^2^, where σ is the standard deviation of the phenotype estimated using the variance of coefficients (Var(β_j_)), minor allele frequency (f_j_), and sample size (n_j_):

2 $$2{n}_{j}{f}_{j}(1-{f}_{j})\sim {\sigma }^{2}\frac{1}{{Var}({\beta }_{j})}-1$$

Here, σ^2^ is the coefficient of the regression, estimating σ such that $\sigma=\sqrt{{\sigma }^{2}}$.

We calculated the posterior probability, π_j_, that the j th variant is driving the association, given l variants in the region, by:

3 $${\pi }_{j}=\frac{(1-\gamma ){\varLambda }_{j}}{l{\sum }_{k=0}^{l}{{{{{\rm{s}}}}}}{\varLambda }_{k}}$$

where γ denotes the prior probability for no association at this locus and k indexes the variants in the region (with k = 0 allowing for the possibility of no association in the region). We set γ = 0.05 to control for the expected false discovery rate of 5%, since we used a threshold of *p* value_marginal_ < 5 × 10^−8^ to identify loci for fine-mapping.

The 95% credible set for each signal was then constructed by (i) ranking all variants according to their Bayes factor and (ii) including ranked variants until their cumulative posterior probability of driving the association attained or exceeded 0.99.

#### Colocalization

We performed colocalization analysis using the coloc method^57^ using default priors and all variants within 500 kb of the index variant. As performed by^57^, we considered two genetic signals to have strong evidence of colocalization if PP3 + PP4 ≥ 0.99 and PP4/PP3 ≥ 5 and suggestive evidence of colocalization if PP3 + PP4 ≥ 0.8 and PP4/PP3 ≥ 3. For gene expression colocalizations, we used summary statistics from GTEx v7^58^. For disease and quantitative trait colocalizations, we used UK Biobank summary statistics of ICD10 codes, normalized quantitative traits (http://www.nealelab.is/blog/2017/7/19/rapid-gwas-of-thousands-of-phenotypes-for-337000-samples-in-the-uk-biobank). For analysis we selected UK Biobank phenotypes where the minimum *p* value within the ±500 kb region around the locus tag SNP was <5 × 10^−8^.

##### Mendelian randomization analysis

The observational studies that are impaired by confounding or reverse causation, it is hard to assess whether the risk factors are causal or correlated with DISH. Mendelian randomization (MR) provides a way of using genetics to test for causal driving factors for a phenotype of interest^59^. In MR, we use genetic variants as a proxy for risk factors to minimize confounding and reverse causation in observational data. Genetic variants are randomly assigned when passed from parents to offspring and therefore minimize the effect of confounders and avoid reverse causation. However, little is known about the genetic component or the causal genes and pathways driving DISH.

There are three important assumptions in MR analyses:

1. the chosen genetic variants are associated with the exposure of interest,
2. they are not associated with any confounders, and
3. they are not associated with the outcome via any pathway other than through the exposure of interest (horizontal pleiotropy)^60^.

We performed two-sample Mendelian randomization analyses using the Inverse Variance Weighted (IVW) or Wald ratio methods included in the R package twoSampleMR. GTEx v7 summary statistics were used for the effect of variants on gene expression of different genes^58^. To identify independent SNP instruments for each exposure, GWAS-significant SNPs (*P* < 5 × 10^−8^) for each risk factor were pruned (*r*^2^ < 0.01; LD window of 250 kb). As a reference panel for LD calculations, we used genotypes from 5,000 UK Biobank participants that were randomly selected after filtering for unrelated European participants. We then estimated the causal effect of the risk factor on the disease trait according to the MR paradigm. The Wald ratio method was used when only one independent variant was associated with expression of the gene in GTEx while IVW was used to test for causal effects in the presence of multiple independent eQTLs for a gene. We applied Bonferroni correction for all the gene-based tests with an FDR cutoff 0.1 to be considered significant. This study was not preregistered.

As a sensitivity analysis, we also ran the MR analysis using MR-Egger regression, weighted median-based, and weighted mode-based tests and tested whether they predicted causal effects of similar direction between exposure and outcome. The weighted median (mode) estimator is the median (mode) of a distribution in which Wald ratio estimates have been ordered and represent percentiles of this distribution^61,62^. The weighted median and mode estimates are less sensitive to the effect of pleiotropic variants as this method assumes that the estimates from pleiotropic variants would be outliers. On the other hand, the MR-Egger approach performs a weighted linear regression of the marginal effect of each SNP to the outcome on the marginal effect of each SNP to the exposure. In this test, the analysis of the regression intercept detects an overall directional pleiotropic contribution of weak instrumental SNPs on the risk estimate^63^.

### Ethics aspects

The UK Biobank project was approved by the National Research Ethics Service Committee North West-Haydock (REC reference: 11/NW/0382). An electronic signed consent was obtained from the participants (more information on UK Biobank participant consent can be found at: https://biobank.ctsu.ox.ac.uk/crystal/crystal/docs/Consent.pdf). UK Biobank data were accessed under the approval of UK Biobank within project 18448. The study was conducted following the principles of the declaration of Helsinki and all participants gave prior written informed consent. All data used in this study were anonymized before its use.

### Reporting summary

Further information on research design is available in the Nature Portfolio Reporting Summary linked to this article.

## Supplementary information

Supplementary Information

Description of Additional Supplementary Files

Supplementary Data 1

Supplementary Data 2

Supplementary Data 3

Supplementary Data 4

Supplementary Data 5

Supplementary Data 6

Reporting Summary

## Data Availability

UK Biobank database is available on request and with permission from UK Biobank (https://www.ukbiobank.ac.uk/). Summary statistics are available from the GWAS catalog under accession number GCST90134532.

## Code availability

Our open source code is freely available at https://github.com/calico/DISH. The version of the code used in this publication is also distributed at 10.5281/zenodo.7796082.
