# Supplementary material for: Genetics implicates overactive osteogenesis in the development of diffuse idiopathic skeletal hyperostosis
Source: Nat Commun. 2023 May 8;14:2644. doi: 10.1038/s41467-023-38279-x (PMC10167361; doi:10.1038/s41467-023-38279-x)
Supplement: Supplementary file 1 — Supplementary Information [file 41467_2023_38279_MOESM1_ESM.pdf]

Supporting Information - Genetics and DISH in UKBB

Supporting Information - Genetics and DISH in UKBB
- Supplementary Figures:
  - Supplementary Figure 1:   Overview of Epidemiological and Genetics Methodology
  - Supplementary Figure 2: Overview of Machine Learning Training and Testing Pipeline
  - Supplementary Figure 3: Human Annotator Validation of Machine Learning Model
  - Supplementary Figure 4: Representative Images of DISH Flow Scores
  - Supplementary Figure 5: Distribution of DISH Scores and Age-dependence
  - Supplementary Figure 6: Association of DISH with Bone Traits
  - Supplementary Figure 7: Sex-stratified skeletal sites BMD DISH Associations.
  - Supplementary Figure 8: Association of DISH with Pain Surveys (Univariate).
  - Supplementary Figure 9: Pre-existing conditions associated with Severity of DISH (Univariate).
  - Supplementary Figure 10: Association of DISH with Plasma Biomarkers and CBC (Univariate).
  - Supplementary Figure 11: Prognostic outcomes associated with DISH flow score
  - Supplementary Figure 12: Comparison of effect size for fine mapped variants on sex-stratified GWAS with the GWAS over the whole imaging cohort.
  - Supplementary Figure 13: Sex-stratified genetic correlations
  - Supplementary Figure 14: PheWAS SNP Table
  - Supplementary Figure 15: PheWAS Gene Manhattan Plots
- Supplementary Note 1: Machine Learning Model
  - Step 1: Identification of anterior intervertebral junctions.
  - Step 2: Scoring the hyperostosis of each intervertebral junction.
  - Step 3: Summing the bridge scores across the spine.
- Supplementary Note 2: Epidemiological Analysis
  - Univariate Associations with Biomarkers and Physiological Risk Factors
  - Regularized LASSO for multivariate variable selection
  - Medical Conditions at Baseline Associated with DISH Flow Score
  - Pain Conditions and Medication Associated with DISH Flow Score
  - Prognostic Outcomes Analysis
- Supplementary References

Supplementary Figures:

Supplementary Figure 1: Overview of Epidemiological and Genetics Methodology

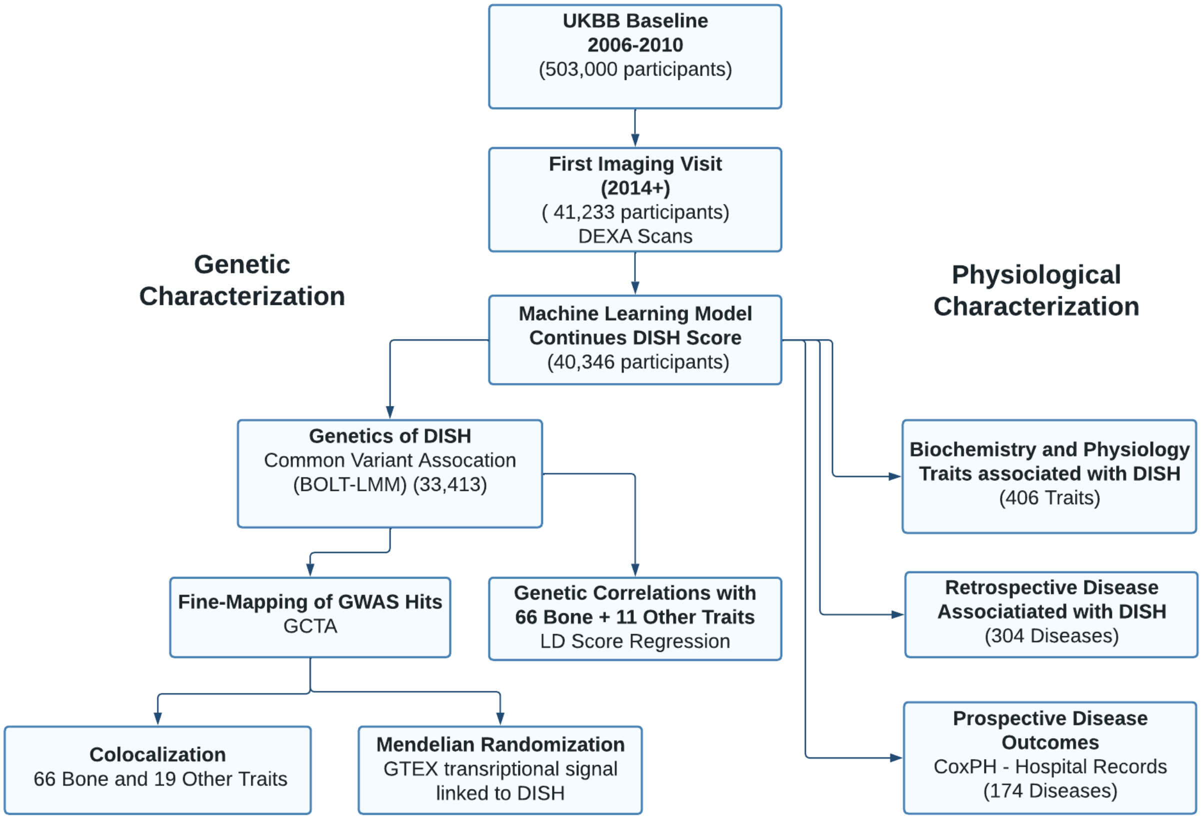

We derived DISH scores (measure of severity of osteophyte formation across spine) using a machine learning model for ~ 40,000 participants with DXA images in UK Biobank Imaging study, The derived scores were used to associate severity of DISH with biomarkers, pre-existing conditions, and future outcomes. We performed genetic characterization of pathology to identify genetic risk factors and genetically correlated traits. For implicated genes, we used GTEX transcriptional expression to test for potential causal associations with DISH.

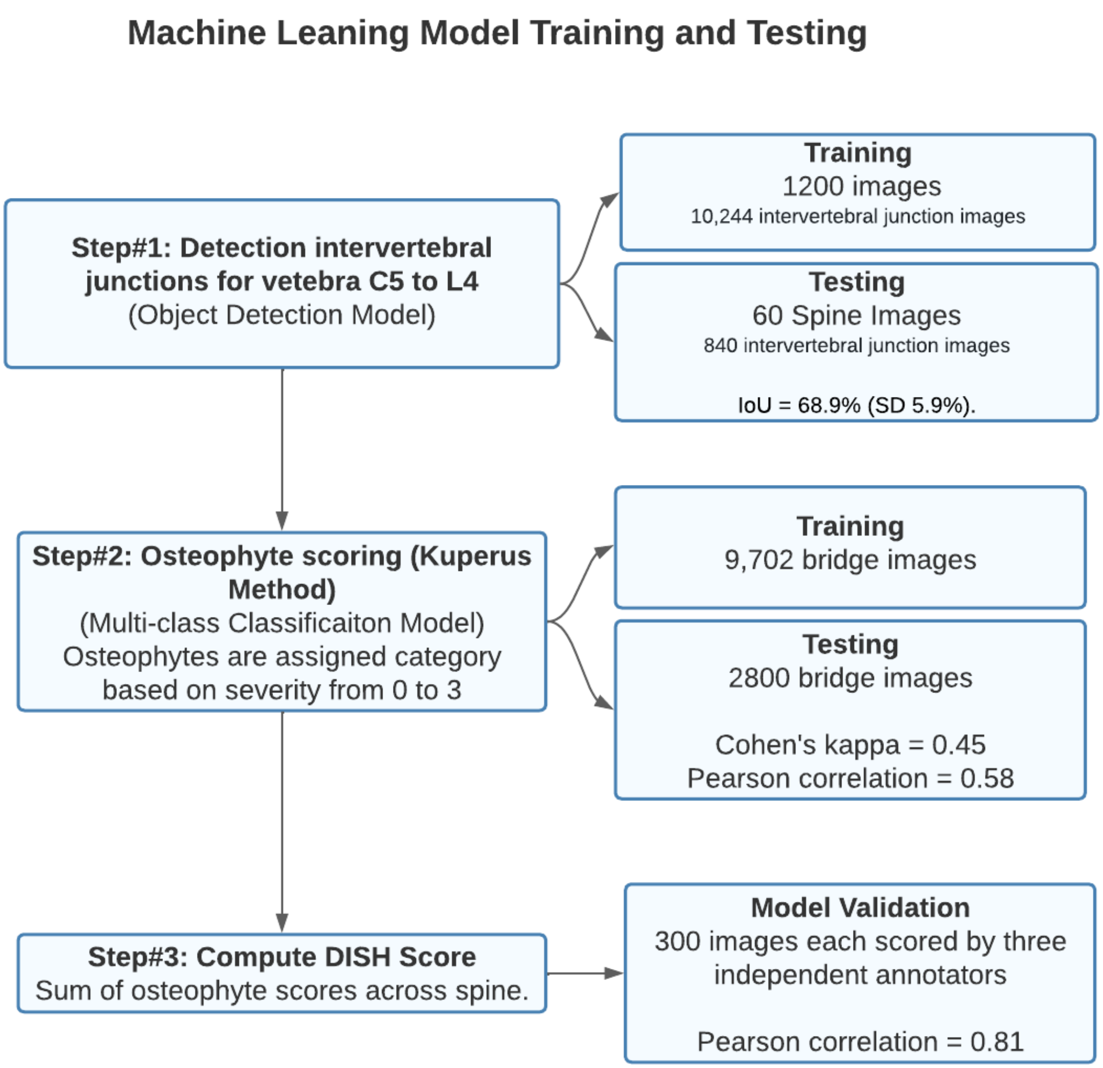

Supplementary Figure 2: Overview of Machine Learning Training and Testing Pipeline
On the left, a schematic diagram outlining the iterative analytical steps in our automated DISH score pipeline. To the right of each step is a description of the training data and test performance for each individual step; for the final step, “Model Validation” indicates the performance of the overall pipeline versus human annotations.

A)

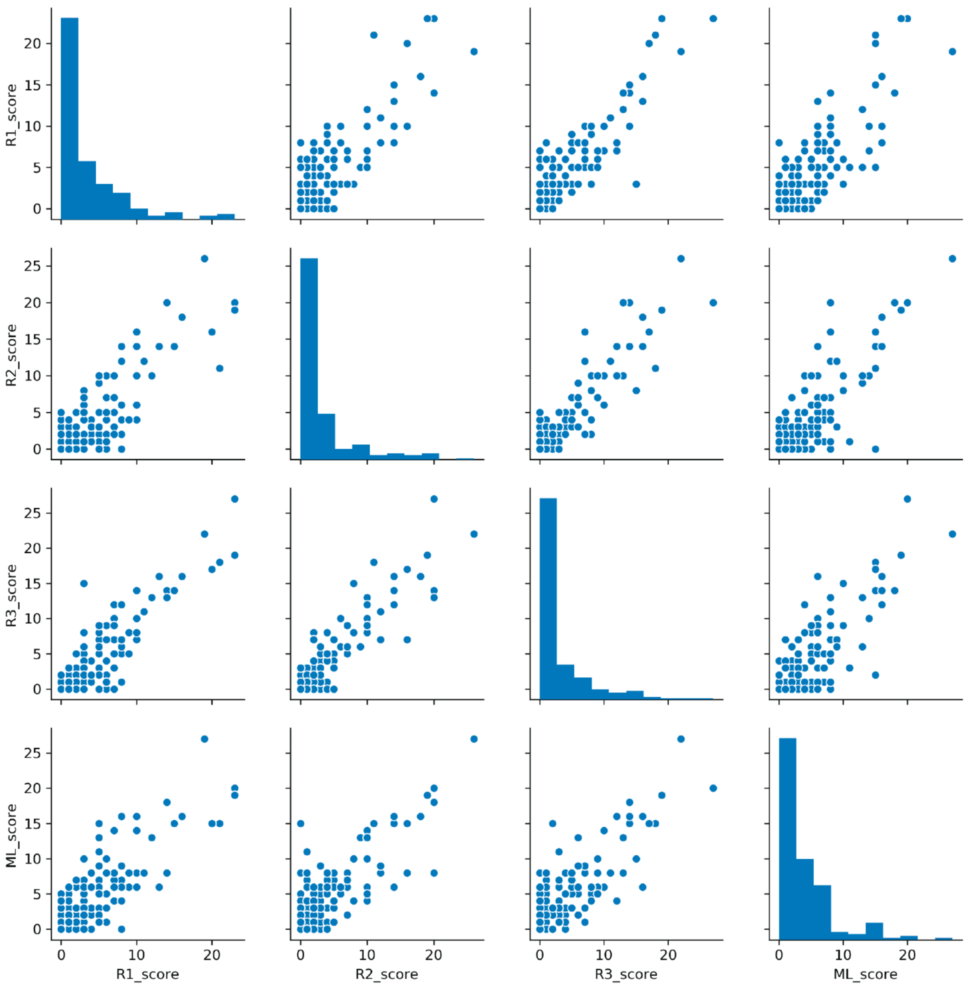

B)

	R1_score	R2_score	R3_score	ML_score
**R1_score**	1.000000	0.841215	0.883528	0.806882
**R2_score**	0.841215	1.000000	0.915644	0.815271
**R3_score**	0.883528	0.915644	1.000000	0.821859
**ML_score**	0.806882	0.815271	0.821859	1.000000

Supplementary Figure 3: Human Annotator Validation of Machine Learning Model

(A) Pairs plot of flow scores between three human annotators (R1, R2, and R3) and the ML model. (B) Pearson correlation coefficients between each annotator and ML model. There is strong agreement (correlation > 0.8) between annotators and machine learning model scores.

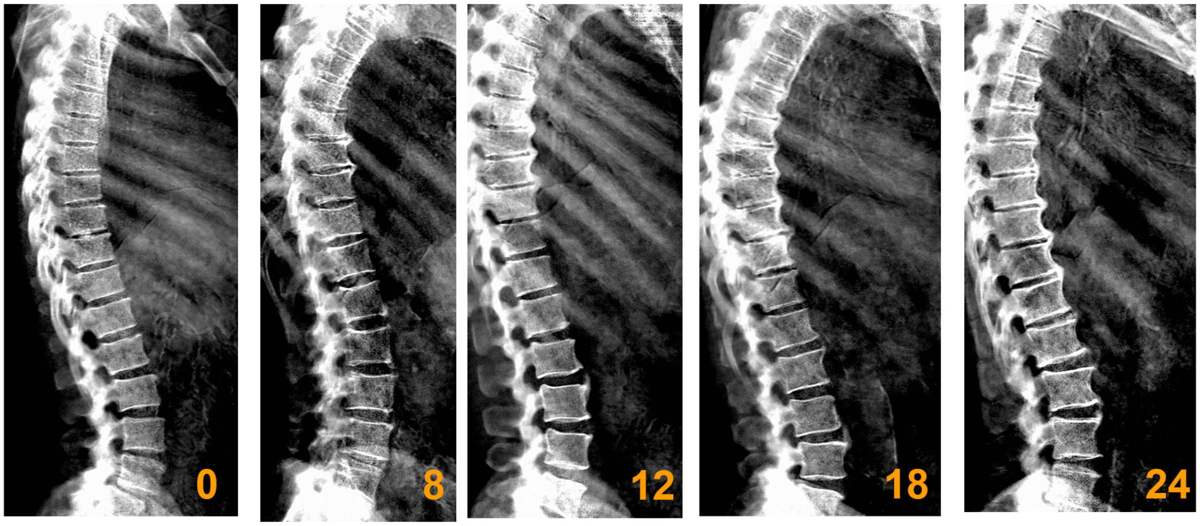

Supplementary Figure 4: Representative Images of DISH Flow Scores

Representative images of flow scores with increasing severity of DISH. Machine learning model assigned each osteophyte a value from 0 to 3 based on the severity of ossification and all scores were summed to create a total score per individual. Scores above 12 are typically consistent with DISH diagnoses based on Resnick criteria.

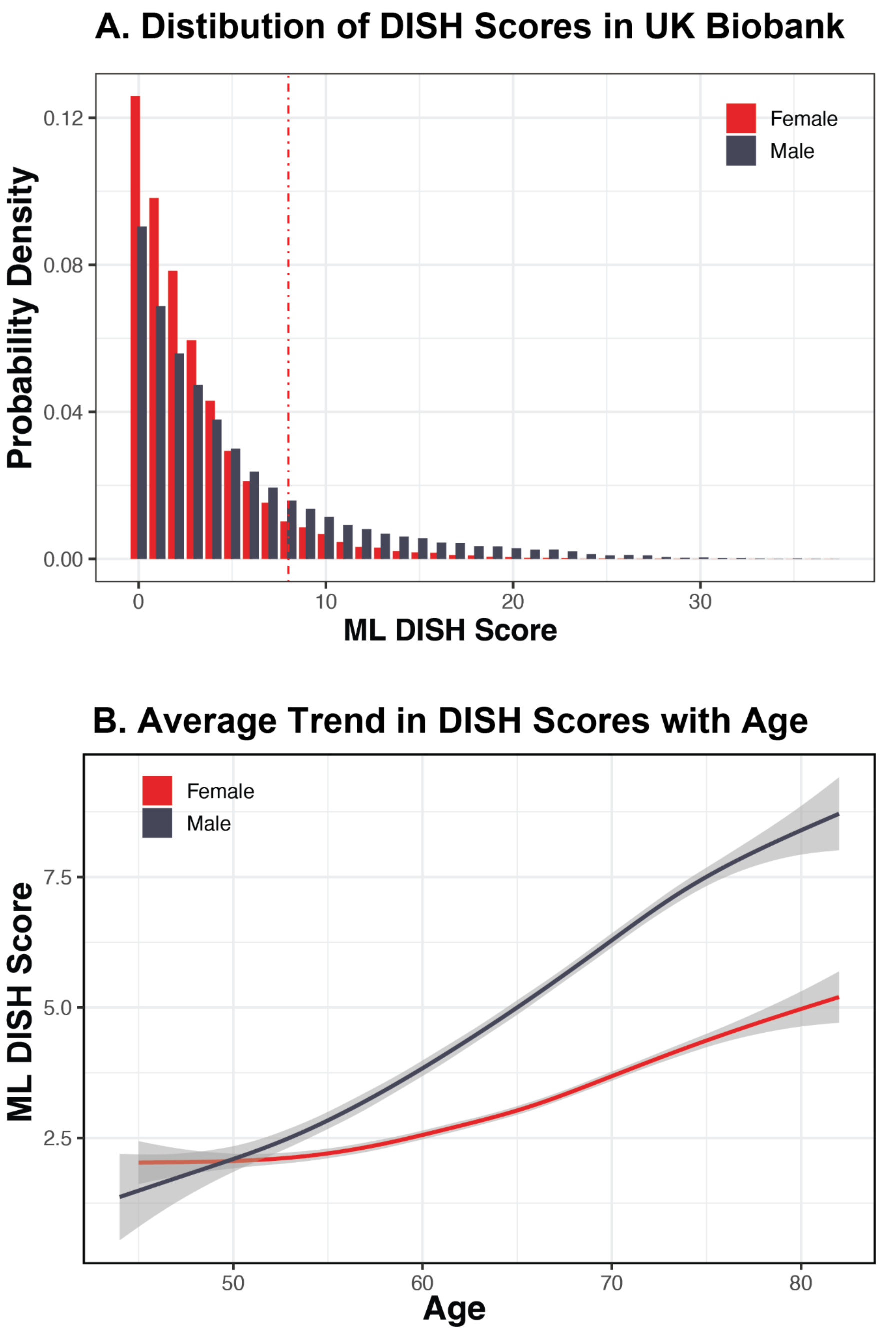

Supplementary Figure 5: Distribution of DISH Scores and Age-dependence

(A) Distribution of DISH Scores across the entire imaging cohort. Flow score above 1 std. dev is shown using a red dashed line.

(B) Increase in DISH Scores as a function of age (sex stratified spline fit). The shaded regions represent the 95% confidence interval of the spline fit.

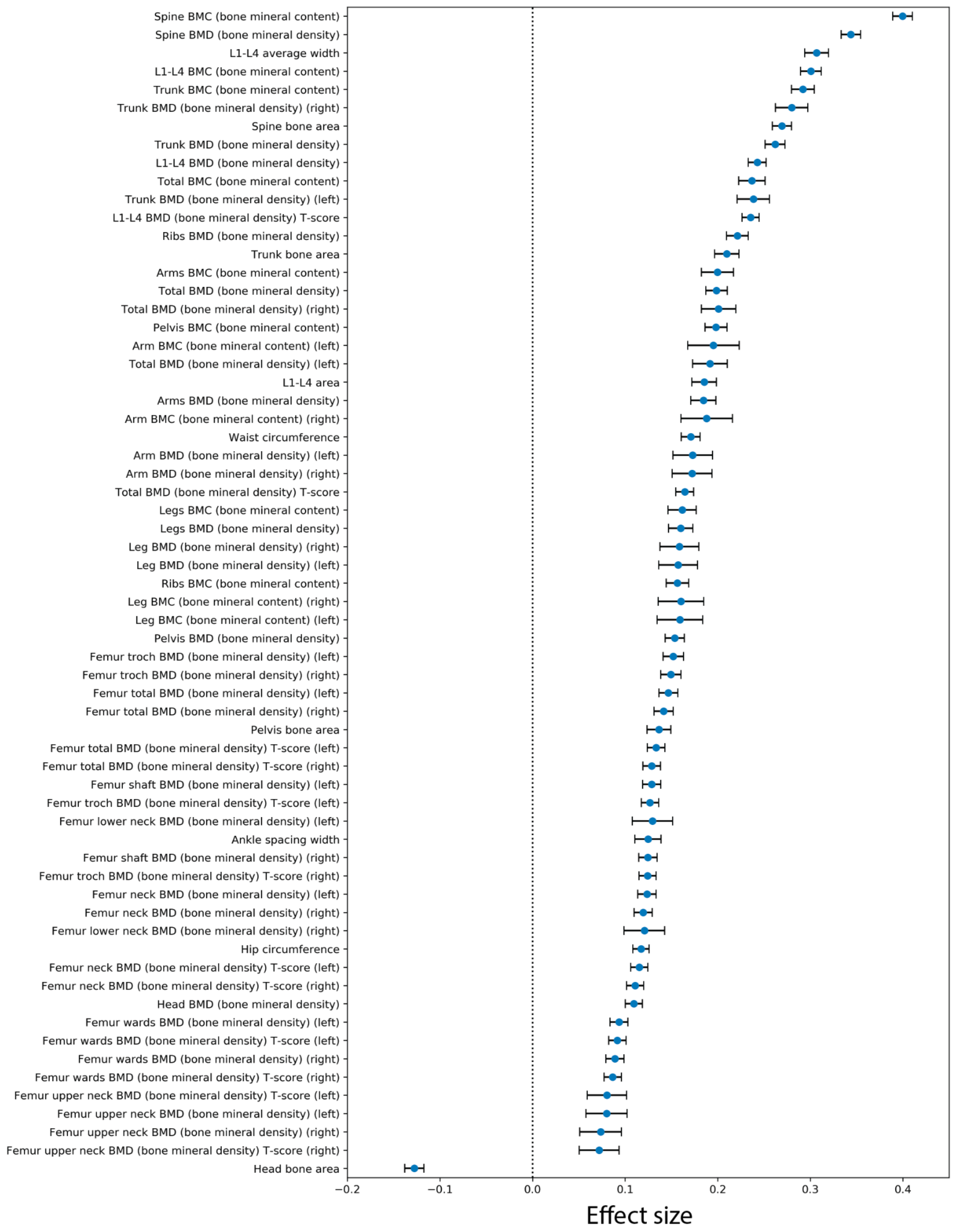

Supplementary Figure 6: Association of DISH with Bone Traits

Univariate associations of bone-traits as a function of Z-scaled DISH flow scores (effect size) tested using linear regression. Only significantly associated bone-traits after multiple hypothesis correction are shown. On average, one standard deviation of DISH flow score (flow score of 4) results in 0.2 standard deviation change in BMC, BMD, and bone area after controlling for age, gender, socioeconomic status, smoking, and ethnicity. The blue dots represent the mean effect size, the error bars represent 95% confidence intervals (n=41,233).

A. Skeletal BMD - DISH Associations

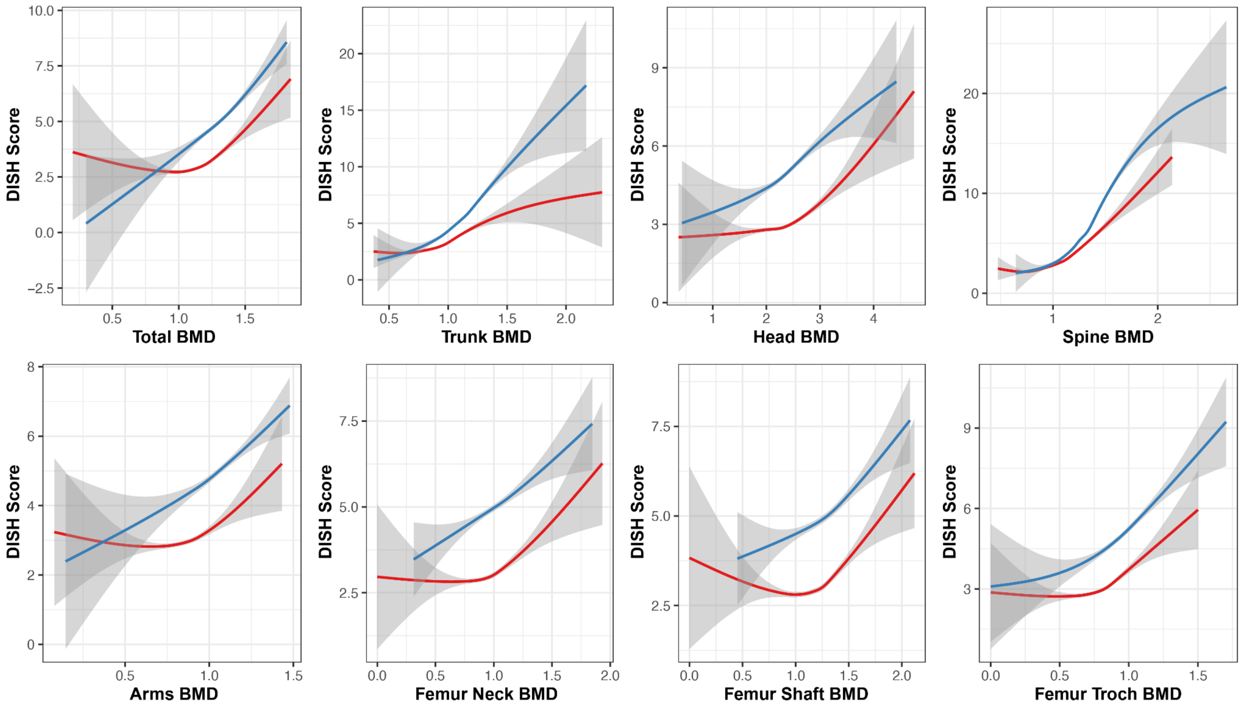

B. Femur BMD - DISH Associations

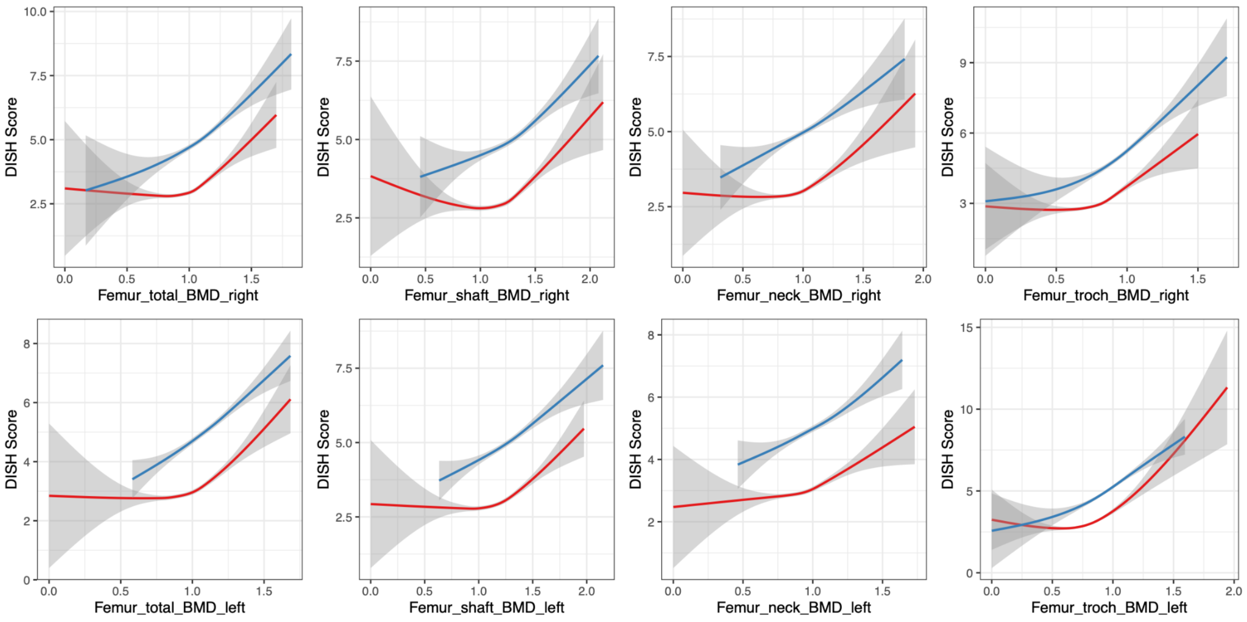

Supplementary Figure 7: Sex-stratified skeletal sites BMD DISH Associations.
Comparison of total, shaft, neck, troch areas on left and right femur. Figure shows sex-stratified spline fit with females(red) and males (blue). Osteophytes sometimes occur in the OA Femur troch area, but are not known to occur in the Femur shaft area. **(Associations are not adjusted for age or other factors).** The shaded regions represent the 95% confidence interval of the spline fit.

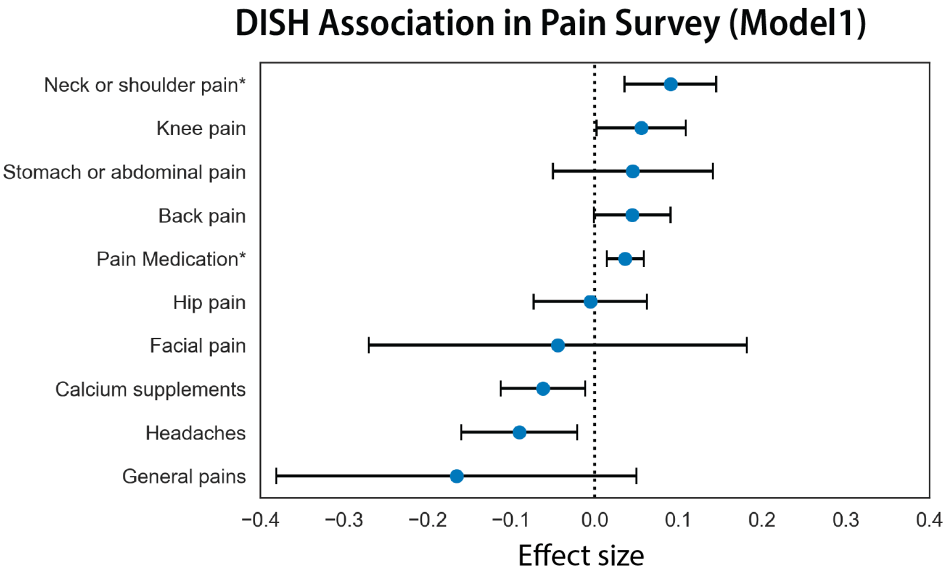

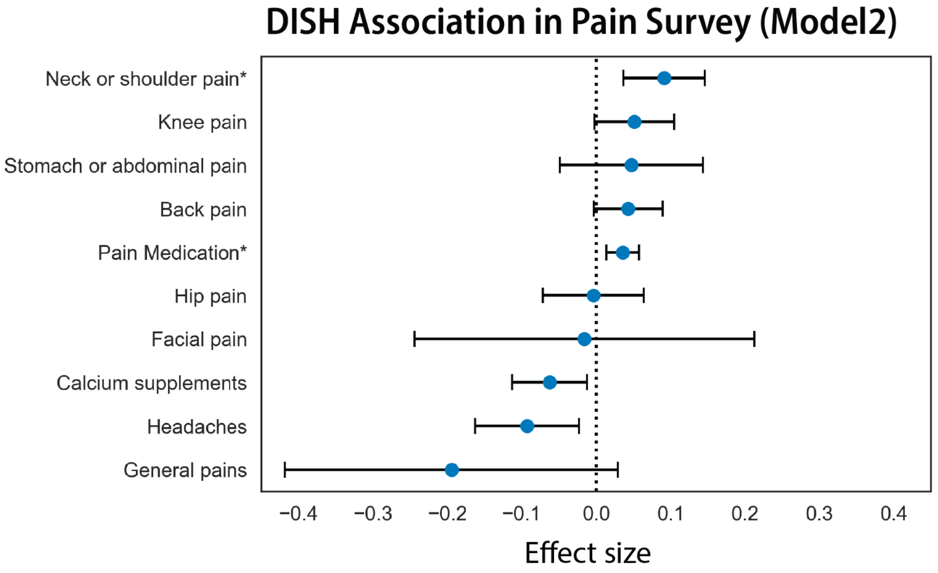

Supplementary Figure 8: Association of DISH with Pain Surveys (Univariate).

Univariate associations of chronic pain (3+ months) in questionnaires with DISH flow score in logistic regression model. Model 1: Pain as function of DISH flow score, age, sex, age*sex, age^2. Model2 = Model 1 + township deprivation index, smoking, ethnicity. DISH scores are Z-scaled. Significant associations after multiple hypothesis correction are marked with “*” on y-axis labels. The blue dots represent the mean effect size (odds ratio), the error bars represent 95% confidence intervals. The number of participants who replied to these questions in the questionnaire during the imaging visit varied widely from 2,360 for stomach pains to 41,233 participants for taking pain medication and the logistic regression is performed with information from all participants who answered the questionnaire.

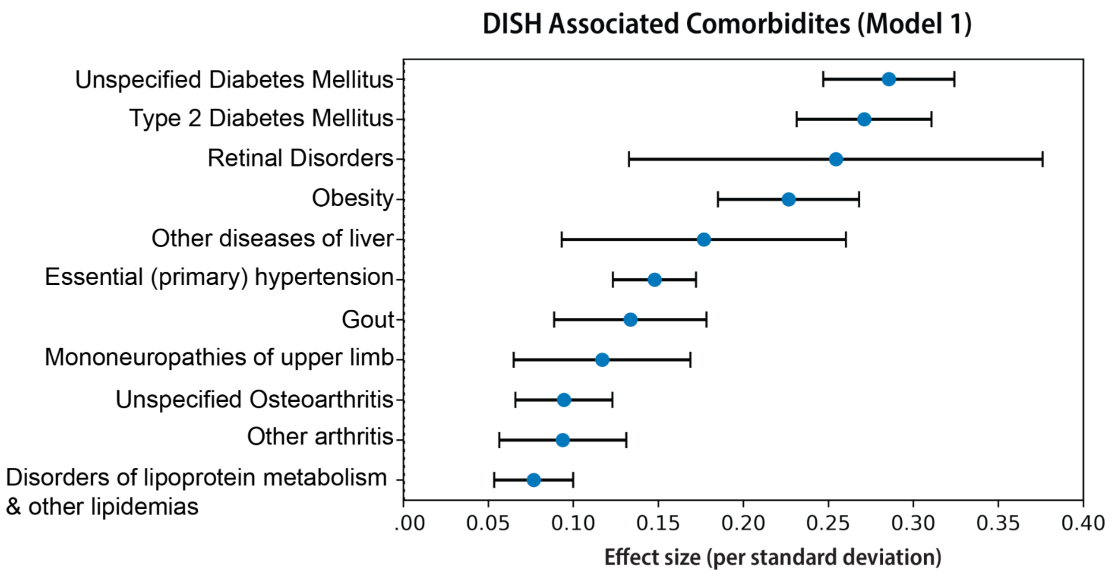

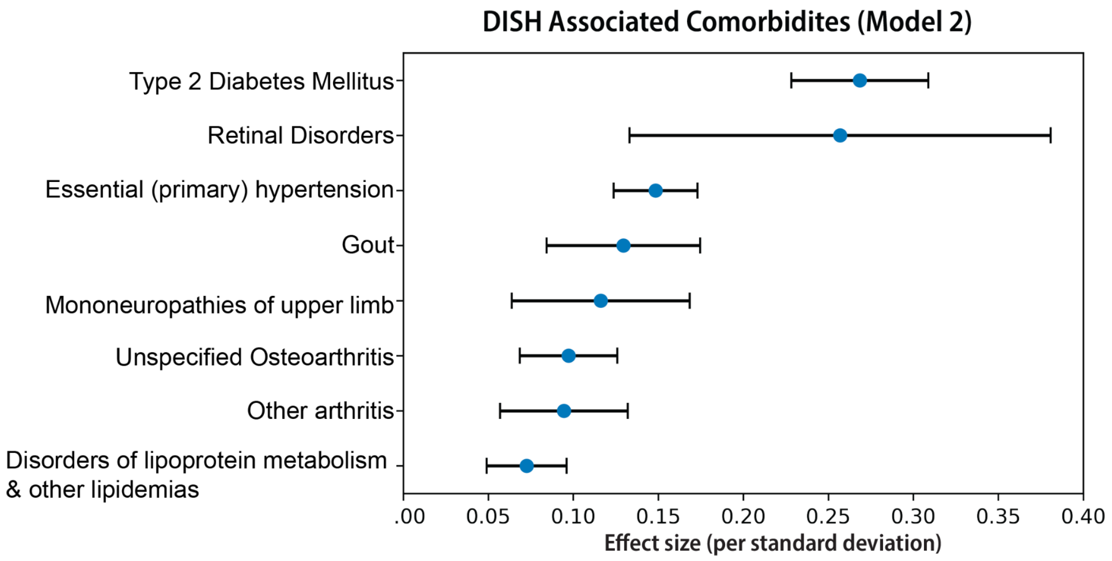

Supplementary Figure 9: Pre-existing conditions associated with Severity of DISH (Univariate).

Univariate association of existing disease with DISH flow score in a logistic regression model. Model 1: Disease is a function of DISH flow score, age, sex, age*sex, age^2. Model2 = Model 1 + township deprivation index, smoking, ethnicity. DISH scores are Z-normalized. 304 diseases were tested. Only significantly associated diseases after multiple hypothesis correction are shown. The blue dots represent the mean effect size, the error bars represent 95% confidence intervals.

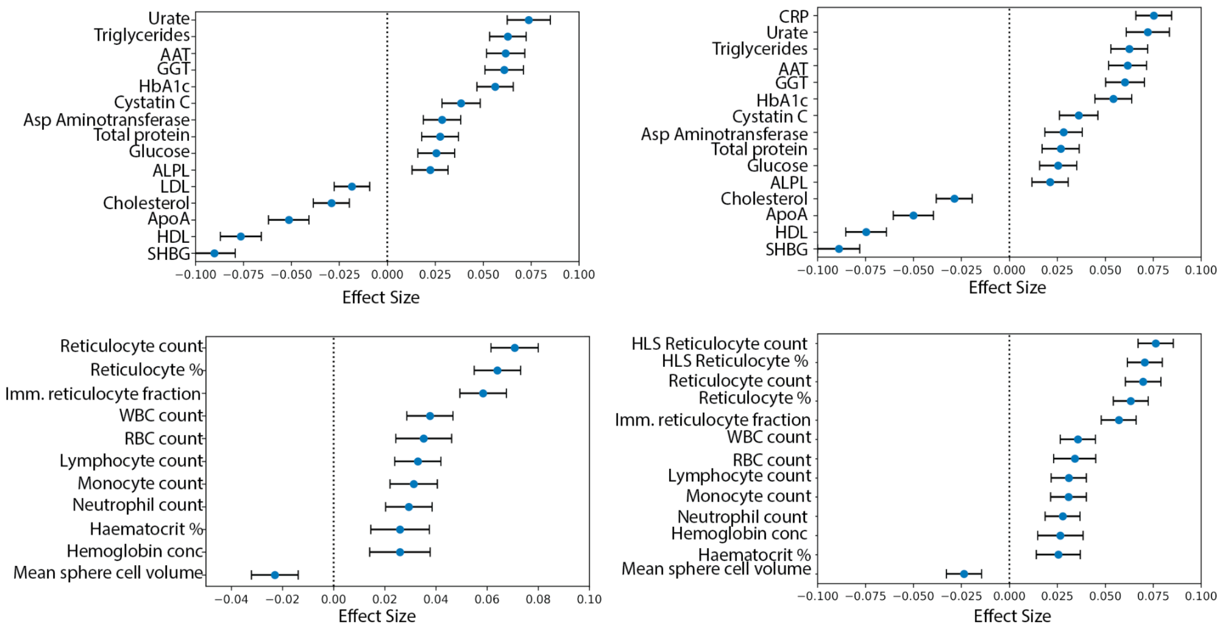

Supplementary Figure 10: Association of DISH with Plasma Biomarkers and CBC (Univariate).

Univariate associations of Biochemistry and CBC measures with DISH flow scores tested using linear regression. Biomarkers are modeled as a function of DISH flow score, age, sex, age*sex, age^2. Model1 is on the right, Model2 is on the left. Model2 = Model 1 + township deprivation index, smoking, ethnicity. DISH scores are Z-normalized. Only significantly associated biomarkers after multiple hypothesis correction are shown. The blue dots represent the mean effect size, the (n=41,233).

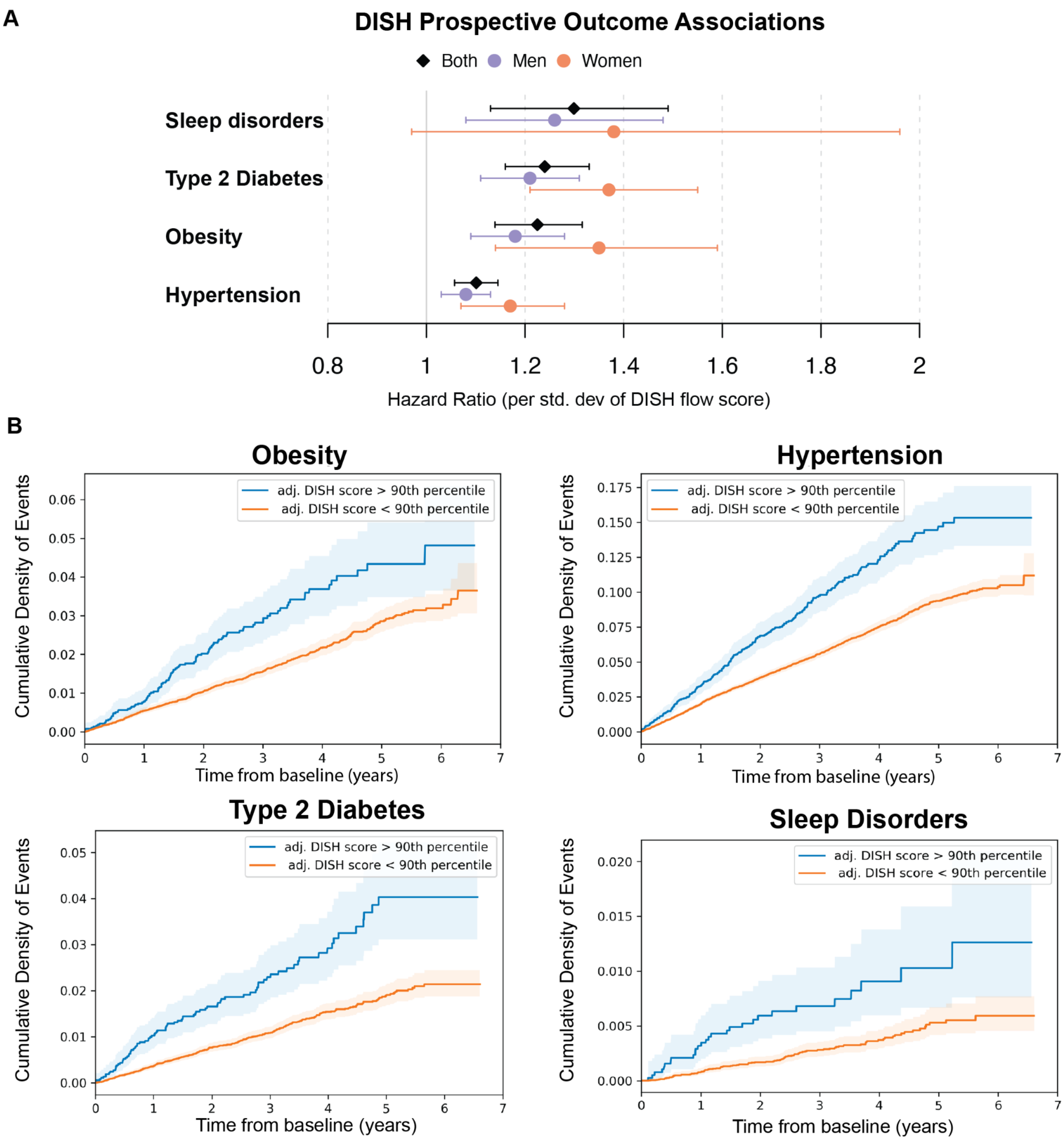

Supplementary Figure 11: Prognostic outcomes associated with DISH flow score

(A) Cox proportional hazards was used to identify the prognostic value of DISH scores and identified four diseases significantly associated with higher DISH scores at baseline (after adjusting for age, gender, socioeconomic status, BMI, and ethnicity). The blue dots represent the

mean hazard ratio, the error bars represent 95% confidence intervals (n=41,233).

(B-F) The cumulative density of events based on stratification by age and sex adjusted DISH scores for the participants. Participants with higher DISH scores at baseline were more likely to be diagnosed with prostate cancer in the future. The shaded regions indicate the 95% confidence interval for each survival curve.

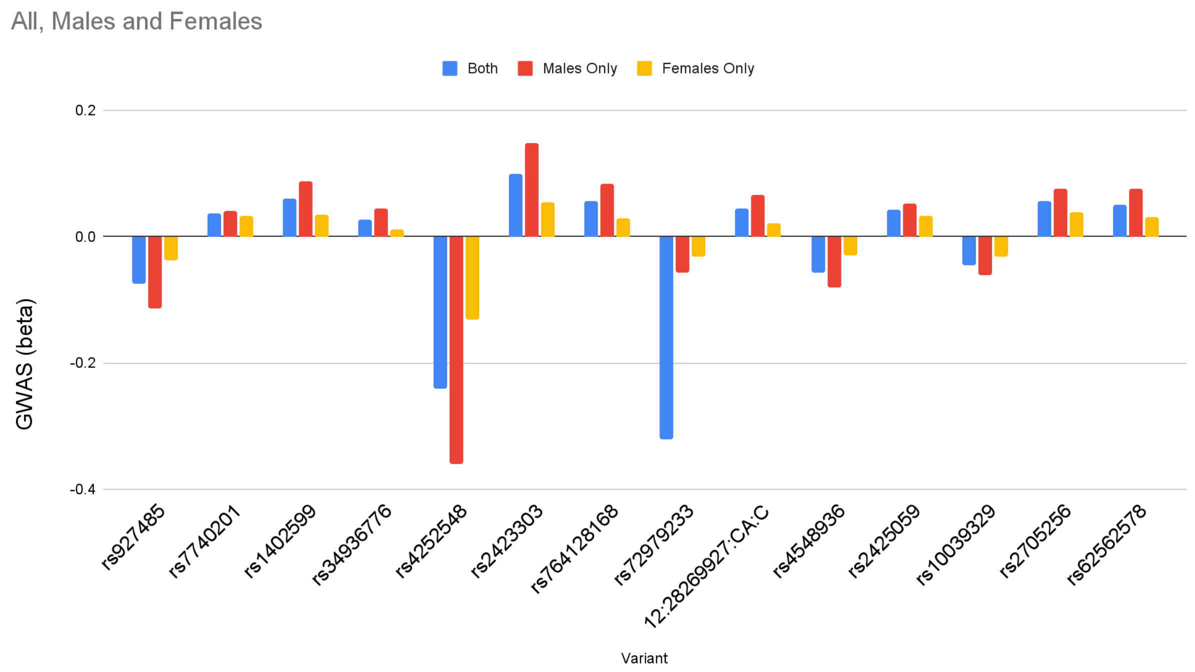

Supplementary Figure 12: Comparison of effect size for fine mapped variants on sex-stratified GWAS with the GWAS over the whole imaging cohort.

The effect sizes of fine mapped variants are compared on all significant loci for GWAS of DISH performed on complete imaging cohort as well as within male and female participants in the imaging cohort.

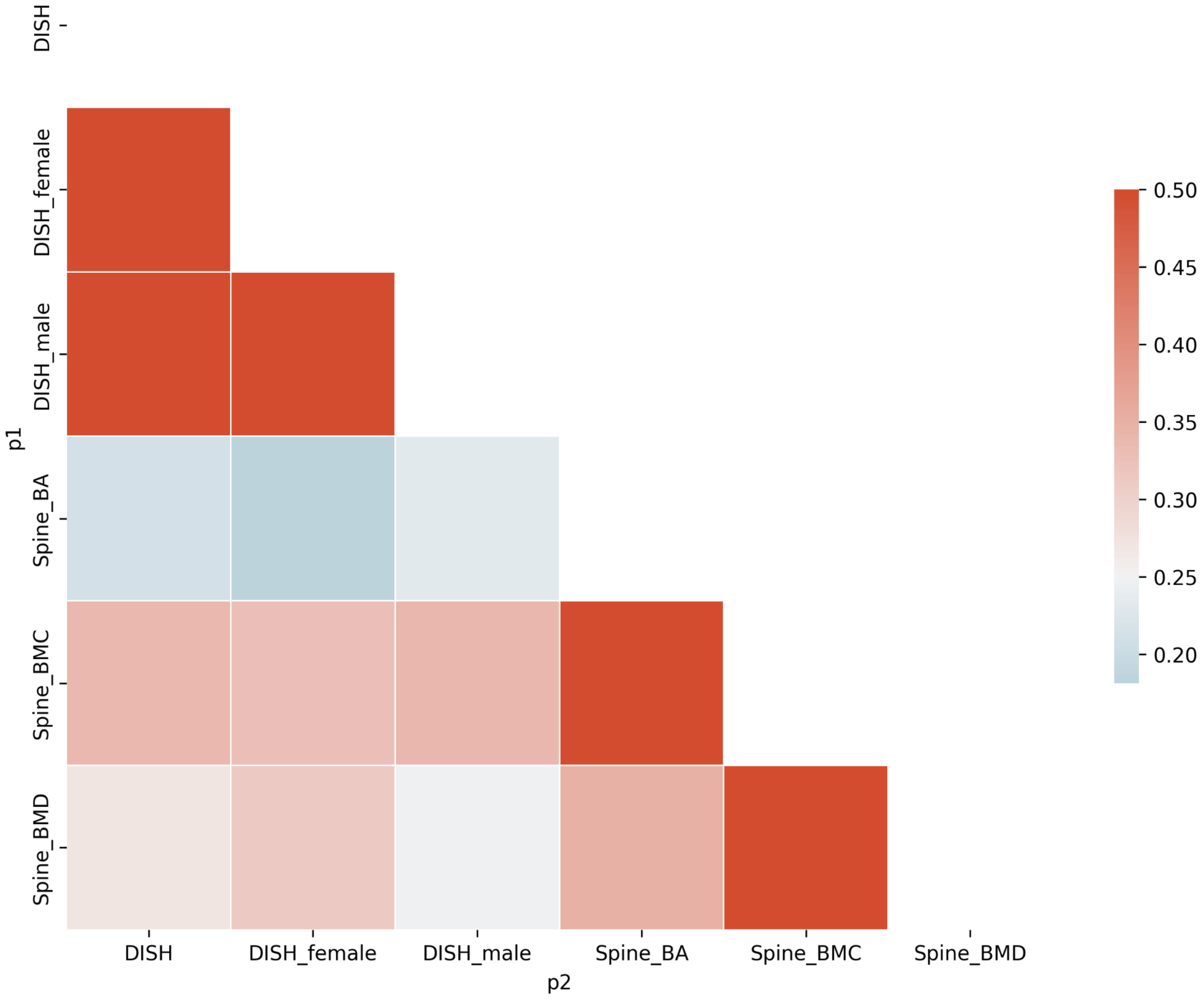

Supplementary Figure 13: Sex-stratified genetic correlations

The correlation between genetic effects determined for sex stratified DISH GWAS and the genetic effects for DISH, Spine BMD (Spine_BMD), Spine BMC (Spine_BMC), and Spine Bone Area (Spine_BA) calculated over the whole cohort. The genetic correlation was calculated using LD-Score regression. The genetic correlation between the sex stratified GWASes for DISH and the non-stratified GWAS of DISH is 0.96. In addition, the genetic correlation between the sex stratified GWASes for DISH and the spine bone traits are also not significantly affected by stratification.

gwasATLAS: Previously Reported GWAS Assocations
(p.value<5e-8)

Domain	Uniq Trait	Population	rs4252548 (IL-11)	rs1402599 (SUPT3H/RUNX2)	rs34936776 (SUPT3H/RUNX2)	rs927485 (SUPT3H/RUNX2)	rs2425059 (UQCC1/GDF15)	rs4548936 (ANKFN1/NOG)	rs62562578 (ROR2)	rs72979233 (POLD3/CHRDL2)
Dermatological	Baldness	UKB2 (EUR)		10.23						
Metabolic	Arm-arm fat ratio	UKB2 (EUR)					8.25			
	Body Mass Index	UKB2 (EUR meta)		7.47						
	Impedance measures - Arm fat-free mass (left)	UKB2 (EUR)	12.94							
	Impedance measures - Arm fat-free mass (right)	UKB2 (EUR)	12.96							
	Impedance measures - Arm predicted mass (left)	UKB2 (EUR)	13.21							
	Impedance measures - Arm predicted mass (right)	UKB2 (EUR)	12.68							
	Impedance measures - Basal metabolic rate	UKB2 (EUR)	11.83							
	Impedance measures - Impedance of leg (left)	UKB2 (EUR)						7.62		
	Impedance measures - Impedance of leg (right)	UKB2 (EUR)						7.33		
	Impedance measures - Leg fat-free mass (left)	UKB2 (EUR)	7.53							
	Impedance measures - Leg fat-free mass (right)	UKB2 (EUR)	8.66							
	Impedance measures - Leg predicted mass (left)	UKB2 (EUR)	7.60							
	Impedance measures - Leg predicted mass (right)	UKB2 (EUR)	8.78							
	Impedance measures - Trunk fat-free mass	UKB2 (EUR)	18.02							7.42
	Impedance measures - Trunk predicted mass	UKB2 (EUR)	17.71							7.33
	Impedance measures - Whole body fat-free mass	UKB2 (EUR)	14.55							
	Impedance measures - Whole body water mass	UKB2 (EUR)	14.62							
	Leg-lef fat ratio	UKB2 (EUR)					21.35			
	Trunk-trunk fat ratio	UKB2 (EUR)					24.52			
Neurological	Corticospinal tract radial diusivities	UKB2 (EUR)		7.49						
Respiratory	FEV2	UKB2 (EUR meta)						15.85		
		UKB2 (EUR)						14.19		
	FVC	UKB2 (EUR meta)						16.06		
		UKB2 (EUR)						12.78		
Skeletal	Bone mineral density	UKB1 (EUR)		11.77				23.52		
	Comparative height size	UKB2 (EUR)	8.94	10.93					7.87	
	Heel bone mineral density	UKB2 (EUR)		36.24	8.10	10.96		40.89		
	Height	AMR+AFR+EAS+Others					9.63			
		EUR		14.72						
		UKB2 (EUR meta)		52.03				17.42		
		UKB2 (EUR)	45.77	26.21				9.19	12.83	12.45
	Osteoarthritis	UKB1 (EUR)					7.63			
		UKB2 (EUR)					10.69			
	Osteoarthritis of hip	UKB2 (EUR)	11.71							
	Osteoarthritis of hip or knee	UKB2 (EUR)					11.08			
	Osteoarthritis of knee	UKB2 (EUR)					15.09			
	Sitting height	UKB2 (EUR)	23.05	15.44					8.30	27.30

Supplementary Figure 14: PheWAS SNP Table

Table of significant associations previously reported in the GWAS Catalog (gwasAtlas) for a set of fine-mapped SNPs linked to DISH. Maximum -log10(p.value) is shown for each trait. Majority of previously reported associations fall into Skeletal and Metabolic categories. The P-values are based on two-tailed distributions to test for association between phenotype and genetic variant.

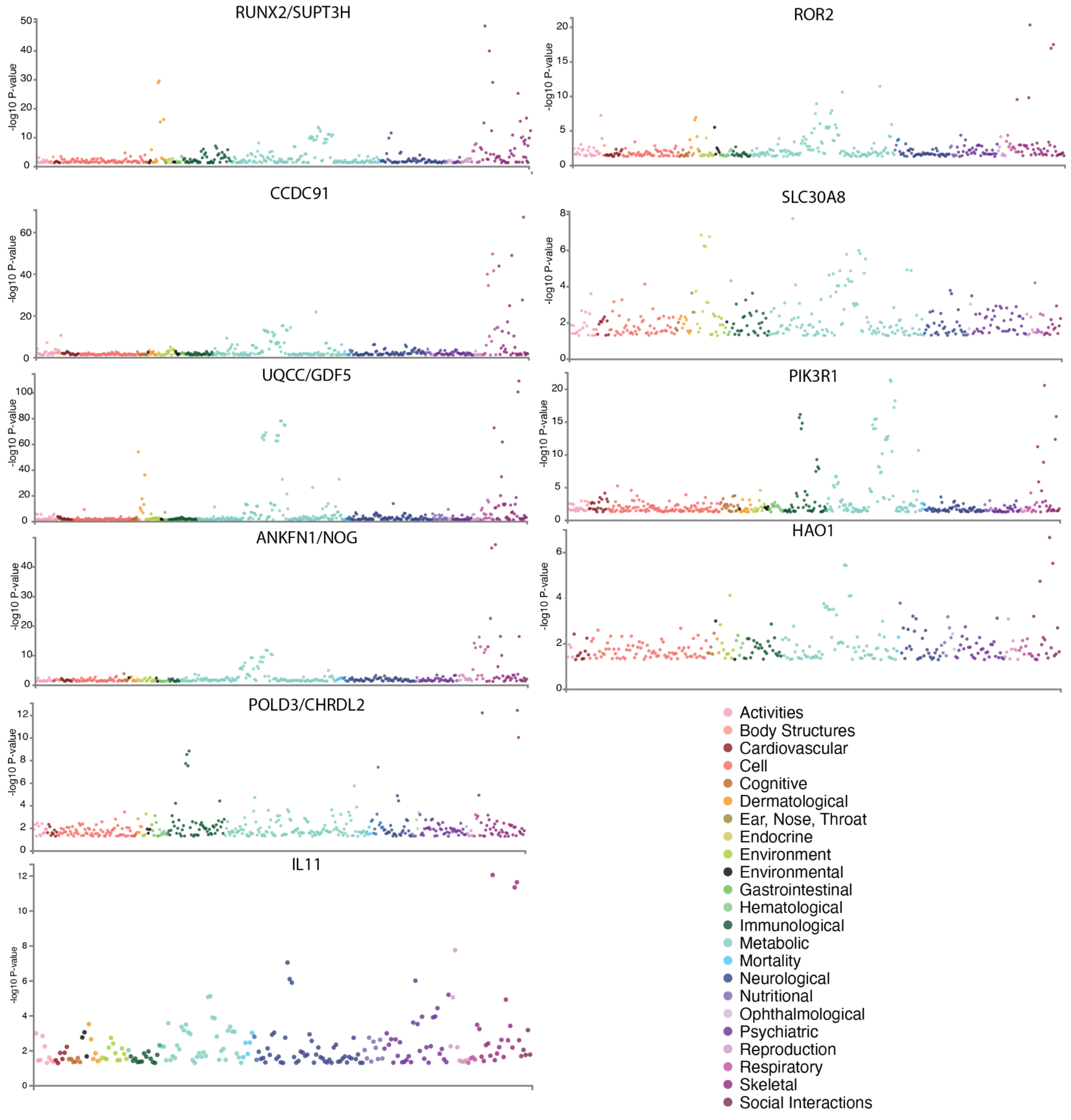

Supplementary Figure 15: PheWAS Gene Manhattan Plots

PheWAS of each of the genetic loci associated with DISH on the GWAS atlas. All of the loci are associated with multiple musculoskeletal traits while some of the loci are more pleiotropic like the GDF5 locus. The P-values are based on two-tailed distributions to test for association between phenotype and genetic variant.

Supplementary Note 1: Machine Learning Model

Step 1: Identification of anterior intervertebral junctions.

Pathological DISH involves the fusing of vertebrae by bony outgrowths that traverse intervertebral gaps. Its pathology results from the summed effects of hyperostosis between all adjacent pairs of vertebrae in the spine. The first step on analysis of DISH was therefore the identification of the anterior portions of the intervertebral gaps along the entire spine. These are the loci where DISH-relevant bridges can form that are visible in lateral DXA images. An object-detection model was applied to this task. It was trained by transfer learning from the MobileNet ssd_mobilnet_v1 model. [1]

A set of 160 images was annotated, which included 2,271 boxes drawn around vertebral junctions. The average number of intervertebral junctions per image (14.2) was used to define the threshold for junction annotation: for each image being evaluated, the 14 highest-confidence annotations returned by the object detector were used.

The annotated images were separated into training and test sets of 100 and 60 images, respectively. Training-set images were augmented by horizontal flipping (all images in the study set are right-facing), inwards adjustment of image borders, brightness, and contrast. In addition, in order to simulate artifacts observed at low frequency across the study set, augmentation was performed by drawing large black or white blocks randomly along the image edges. The final augmented training set included 1200 images and 10,244 boxes.

Performance of the object detector was evaluated in the 60-image test set using intersection-over-union (IoU) for the 14 top-scoring predicted boxes versus all of the annotated boxes, allowing each predicted box's intersection to only be counted for its most-overlapping annotated counterpart. The average IoU across the 60 test images was 68.9% (SD 5.9%).

Step 2: Scoring the hyperostosis of each intervertebral junction.

For each intervertebral junction, a numeric score was to be assigned according to the criteria described in Figure 2 of Kuperus et al (2018). Kuperus et al provide examples and descriptions of hyperostosis between adjacent vertebral bodies, scored on a 0-3 scale in terms of both "bridge" and "flow". We automated that scoring, with greater attention paid to the "Bridge score" than the "Flow score" scale, using an image classification model. This model classified images of individual bridges, i.e. images extracted from the source image using the 14 top-scoring boxes, defined by the object detection model described above. Four categories were established and named

numerically with reference to the bridge score ("br0", "br1", "br2", and "br3"), corresponding to the severity of hyperostosis.

For the training and testing of this image classification model, the object detection model was used to draw boxes (top-scoring 14 per image) across 893 DXA spine images. Each of the resulting 12,502 box images was manually classified as described above. For the test set, 200 of the DXA images (comprising 2800 bridge images) were randomly selected; the remaining 693 DXA images (9,702 bridge images) made up the pre-augmentation training set. The categories (named "br0", "br1", "br2", and "br3", corresponding to the bridge scores) were not evenly balanced (shown for the total annotation set):

Class	Count	%
Br0	10270	82.15
br1	1740	13.63
br2	356	2.85
br3	172	1.38

For the training set, the full data set was augmented first using a horizontal flip.
In the following augmentation steps, imbalance between the classes was reduced by down-sampling from the "br0" and "br1" classes (including in the selection of non-augmented boxes). For each augmentation step, a separate randomly-selected subset of the available boxes (bridge images) was sampled, ensuring maximum diversity of images but nonetheless consistent proportions of augmentation treatments across the classes. The use of only 10% of "br0" boxes and 25% of "br1" boxes resulted in the following proportions:

Class	Input%	Sampled%	Final%
br0	82.15	10	51.8
br1	13.63	25	21.5
br2	2.85	100	18.0
br3	1.38	100	8.7

Bridge images were extracted during the augmentation process, allowing the box itself to be randomly modified. The following augmentation combinations were performed: 1) non-augmented; 2) random tilt up to 30 deg.; 3) random adjustment of the box edge positions by up to 20% of the box width or height; 4) tilt & edge; 5) tilt & brightness; 6) edge & brightness; 7) tilt & contrast; 8) edge & contrast. Augmentation therefore increased the training set size by 8-fold, resulting in the following counts for bridge images by class:

Class	Count
Br0	12752
br1	5272
br2	4496
br3	2112

Training was performed using transfer learning from the efficientnet/b1 model. Evaluated using the test set described above, the Cohen's kappa value for the final model was 0.405 with the following confusion matrix (rows=human, cols=model):

	br0	br1	br2	br3	total
br0	2102	194	31	65	2300
br1	195	171	31	40	385
br2	8	19	29	26	75
br2	1	6	5	33	40
total	2306	234	96	164	

Due to the numeric nature of the classes, the model was also evaluated against the test set using Pearson correlation (using the numeric values of each class "br0", "br1", "br2", and "br3"), and the Pearson correlation against the test set was 0.581.

Step 3: Summing the bridge scores across the spine.

The final output value of the model evaluates overall DISH-like hyperostosis across the spine. Final evaluation was performed using a hold-out set of 200 DXA images that were scored by three independent raters (evaluation was performed using the mean rater score for each DXA image). Those raters used the same bridge-score scheme described above, with the appearance of DISH-related bony outgrowth scored as either a 1, 2 or 3 (bridges without observable outgrowth implicitly received a score of 0). For each DXA image, those numeric scores were summed to produce the final DISH score.

In addition to the final hold-out test used for model evaluation, the independent rater also produced a training set of 199 images (Rater Training) that were used to compare alternative ML models and alternative strategies for interpretation of the ML model output. The classification model's test set annotations were used ensemble across each DXA image for the same purpose (Preliminary Training).

In the case of Rater Training, performances of the object-detection and classification models were being evaluated simultaneously. In the case of Preliminary Training, only the performance of the classification model (and the interpretation of its output) were being evaluated.

For each DXA image, the top-scoring 14 boxes from the object-detection model were used to define sub-images that were scored by the classification model, both described above. Initially, the numbers associated with the class assigned to each of the 14 bridge images ("br0", "br1", "br2", "br3") were summed to produce the model-derived DISH score. Two modifications were added to this process, described below.

First, bridges assigned a score of 1 ("br1") were re-evaluated and assigned a decimal score in the interval [0-1]. That value was calculated as the fraction of confidence scores assigned by the model to classes "br1", "br2", and "br3". This had the general effect of down-weighting "br1" assignments, which frequently were made spuriously (see the confusion matrix above), unless they looked more like "br2"/"br3" instances (which provide a rare source for mis-classification) than they looked like "br0" instances (which provide an abundant source for mis-classification). This modification is referred to below as the "augmentation of one" (Aug.One).

Second, the training of both models on horizontally-flipped images, despite the invariance of right-facing images in the study set for which this tool was being developed, allowed the implementation of a horizontal-flip data augmentation strategy during evaluation. Each DXA image was scored twice: once in its original orientation, once in its horizontally-flipped orientation. The output score was taken as the average of those two scores. This allowed the impact of both models' idiosyncrasies to be minimized. This modification is referred to below as "Aug.Flip".

Pearson correlation coefficients:

Modification	Prelim. Tr.	Rater Tr.
None	0.832	0.821
Aug.One	0.824	0.834
Aug.Flip	0.839	0.838
Aug.One + Aug.Flip	0.828	0.850

Both Aug.One and Aug.Flip were used for the final application of the model.

Supplementary Note 2: Epidemiological Analysis

Univariate Associations with Biomarkers and Physiological Risk Factors

We used linear regression to examine the association of DISH flow score with various biomarkers and physiological markers. For these models, univariate associations of DISH flow score with each marker were evaluated with two different models. In model 1, all univariate associations were performed after adjusting for age, age2, sex, and age x sex. In model 2, we also adjusted for Townsend deprivation index, smoking status, and self- reported primary ethnicity in addition to age, age2, sex, and age x sex. We chose these factors based on their associations with traditional disease outcomes and/or their association with DISH flow score. All p-values were calculated using the two-tailed t-statistic of the estimated association. The associations were considered to be significant after multiple hypothesis testing (i.e., Bonferroni Correction with p-value < 1.1e-4). These estimations were calculated using the statsmodels package v.0.9.0 in python [2].

Regularized LASSO for multivariate variable selection

LASSO is a regularized approach to identify all the relevant variables associated with survival in a single iteration [3]. In this approach, the DISH flow score was modeled as a multivariate linear regression with all biomarkers and physiological risk factors measured in UKBB as covariates. The coefficients (α_j) that relate each covariate (x_j) to the DISH flow score (y_j) are assumed to have regularized Laplace priors. The hyperparameters on the Laplace distribution are also learned from the data:

$$\alpha_j = L(0,\ \lambda)$$
$$\lambda = HC(0,\ 1)$$

$$y_j = \sum_j \alpha_j x_j$$

where $L(0,\ \lambda)$represents the Laplace distribution with location at 0 and λ is the scale parameter while $HC(0,\ 1)$represents the Half Cauchy distribution with location at 0 and a scale parameter of 1.

We utilized Markov Chain Monte Carlo (MCMC) simulations [4] with the No U-Turn Sampler (NUTS) [5] to infer the parameters given the structure of the network. Each simulation was run for 1000 steps with 1000 steps of burn in time and 4 chains were run per simulation to check for convergence of the simulations. During the simulation, a variable is selected if the 95% credible interval of the corresponding coefficient does not overlap with 0. All Bayesian inference was performed using the pymc3 module [6].

Medical Conditions at Baseline Associated with DISH Flow Score

We also evaluated the association of different pre-existing conditions with DISH flow scores in the UKBB. The 10th edition of the international classification of diseases (ICD10) diagnosis codes and date of initial diagnoses were extracted from a combination of general practitioner, electronic health records, and questionnaires filled by all participants when they visited the center. We used the date of visiting the UKBB center to identify a participant's pre-existing conditions. We used logistic regression to examine the association of DISH flow score with pre-existing medical conditions. For these models, univariate associations of DISH flow score with each diagnosis code were evaluated with two different models. In model 1, all univariate associations were performed after adjusting for age, age^2, sex, and age x sex. In model 2, we also adjusted for Townsend deprivation index, smoking status, and self- reported primary ethnicity in addition to age, age^2, sex, and age x sex. We chose to test for association with 304 most prevalent diseases in UKBB . The associations were considered to be significant after multiple hypothesis testing (i.e., Bonferroni Correction with p-value < 1.6e-4).

Pain Conditions and Medication Associated with DISH Flow Score

Finally, we evaluated the association between DISH flow score and replies to pain questionnaires in the UKBB. In particular, we focused on the replies to chronic pain in different regions of the body as well as whether the participant was taking pain medication and calcium supplements regularly. The number of participants who replied to these questions in the questionnaire during the imaging visit varied widely from 2,360 for stomach pains to 43481 participants for taking pain medication.   We used linear regression to examine the association of DISH flow score with pre-existing medical conditions. For these models, univariate associations of DISH flow score with each marker were evaluated with two different models. In model 1, all univariate associations were performed after adjusting for age, age^2, sex, and age x sex. In model 2, we also adjusted for Townsend deprivation index, smoking status, and self- reported primary ethnicity in addition to age, age^2, sex, and age x sex. We tested the association of DISH with 10 different fields in the questionnaire and the associations were considered to be significant after multiple hypothesis testing (i.e., Bonferroni Correction with p-value < 5e-3).

Prognostic Outcomes Analysis

We used Cox proportional hazards models to examine the association of DISH with various diseases. The 10th edition of the international classification of diseases (ICD10) diagnosis codes and date of initial diagnoses were extracted from electronic health records of all participants. The ICD10 codes are hierarchically organized and were created for insurance billing purposes. The PheWAS codes attempt to group the different ICD10 codes into medically meaningful groups [7]. We converted the ICD10 codes at level 2 hierarchy into Phecodes before associating with predicted DISH [8]. Any participant diagnosed with the Phecode prior to baseline was removed from analysis before calculating associations with DISH scores. All cancer events were considered including those reported in the death certificate. We define the baseline to be the date of the first imaging visit (2014+) at the assessment center. Follow up time is defined as time to the first disease diagnosis. If an individual has not developed a pathology or has died before January-2021 they are censored.

We evaluate associations of DISH with each disease using two different Cox proportional hazards models. In model 1, all univariate associations were performed after adjusting for age and sex, while in model 2, we also adjusted for BMI, Townsend deprivation index, and race in addition to age and sex. All p-values were calculated using the two-tailed t-statistic of the estimated association. These estimations were calculated using the statsmodels package v.0.9.0 in python [2]}. We tested 180 PheWAS codes for prognostic association with calcification and only associations that passed multiple hypotheses testing in model 1 (Bonn Ferroni corrected p-value < 2.8e-4) were considered significant.

Supplementary References

1. Howard, A. G. *et al.* MobileNets: Efficient Convolutional Neural Networks for Mobile Vision Applications. *arXiv [cs.CV]* (2017).
2. Seabold, S. & Perktold, J. Statsmodels: Econometric and statistical modeling with python. *of the 9th Python in Science Conference* (2010).
3. Tibshirani, R. Regression shrinkage and selection via the lasso. *J. R. Stat. Soc.* **58**, 267–288 (1996).
4. Gilks, W. R., Richardson, S. & Spiegelhalter, D. *Markov Chain Monte Carlo in Practice*. (CRC Press, 1995).
5. Hoffman, M. D. & Gelman, A. The No-U-Turn Sampler: Adaptively Setting Path Lengths in Hamiltonian Monte Carlo. *arXiv [stat.CO]* (2011).
6. Salvatier, J., Wiecki, T. V. & Fonnesbeck, C. Probabilistic programming in Python using PyMC3. *PeerJ Computer Science* vol. 2 e55 Preprint at https://doi.org/10.7717/peerj-cs.55 (2016).
7. Denny, J. C. *et al.* Systematic comparison of phenome-wide association study of electronic medical record data and genome-wide association study data. *Nat. Biotechnol.* **31**, 1102–1110 (2013).
8. Wu, P. *et al.* Mapping ICD-10 and ICD-10-CM Codes to Phecodes: Workflow Development and Initial Evaluation. *JMIR Med Inform* **7**, e14325 (2019).
