# Supplementary material for: Genetics implicates overactive osteogenesis in the development of diffuse idiopathic skeletal hyperostosis
Source: Nat Commun. 2023 May 8;14:2644. doi: 10.1038/s41467-023-38279-x (PMC10167361; doi:10.1038/s41467-023-38279-x)
Supplement: Supplementary file 2 — Description of Additional Supplementary Files [file 41467_2023_38279_MOESM2_ESM.pdf]

Description of Additional Supplementary Files

File Name: Supplementary Data 1
Description: Comparison between ML Scores and Binarized DISH Diagnosis based on Resnick Criteria. Two expert annotators annotated whether a person could be diagnosed with DISH based on imaging data.

File Name: Supplementary Data 2
Description: The prevalence of DISH in the population stratified by age and sex. Prevalence is tabulated as a function of DISH score threshold.

File Name: Supplementary Data 3
Description: Counts of Existing DISH Diagnosis in UKBB based on being diagnosed with relevant ICD10 codes in electronic health records.

File Name: Supplementary Data 4
Description: Comparison of effect sizes, P-values, and effect allele frequency (EAF) for finemapped variants on sex-stratified GWAS with the GWAS over the whole imaging cohort. In general, due to reduced variability of DISH in females, we observed no genome wide significant associations between DISH and genetics in females while the genome wide significant associations for DISH in men reduced due to smaller n. However, the effect sizes for genetic associations are in the same direction across both males and females in the sex stratified GWAS. In addition, we also show that the effect allele frequency of the fine mapped variants are similar in males and females within the imaging cohort.

File Name: Supplementary Data 5
Description: Heritability of bone traits measured in UKBB using DXA scans. The heritability is estimated using REML and refers to estimated narrow sense heritability.

File Name: Supplementary Data 6
Description: Heritability of comorbid and relevant clinical phenotypes measured in UKBB using DXA scans. The heritability is estimated using REML and refers to estimated narrow sense heritability.
