# Supplementary material for: Genetics implicates overactive osteogenesis in the development of diffuse idiopathic skeletal hyperostosis
Source: Nat Commun. 2023 May 8;14:2644. doi: 10.1038/s41467-023-38279-x (PMC10167361; doi:10.1038/s41467-023-38279-x)
Supplement: Supplementary file 9 — Reporting Summary [file 41467_2023_38279_MOESM9_ESM.pdf]

nature portfolio

Corresponding author(s): Anurag Sethi and Eugene Melamud

Last updated by author(s): Apr 3, 2023

Reporting Summary

Nature Portfolio wishes to improve the reproducibility of the work that we publish. This form provides structure for consistency and transparency in reporting. For further information on Nature Portfolio policies, see our Editorial Policies and the Editorial Policy Checklist.

Statistics

For all statistical analyses, confirm that the following items are present in the figure legend, table legend, main text, or Methods section.

n/a	Confirmed	
☐	☒	The exact sample size (*n*) for each experimental group/condition, given as a discrete number and unit of measurement
☐	☒	A statement on whether measurements were taken from distinct samples or whether the same sample was measured repeatedly
☐	☒	The statistical test(s) used AND whether they are one- or two-sided<br>*Only common tests should be described solely by name; describe more complex techniques in the Methods section.*
☐	☒	A description of all covariates tested
☐	☒	A description of any assumptions or corrections, such as tests of normality and adjustment for multiple comparisons
☐	☒	A full description of the statistical parameters including central tendency (e.g. means) or other basic estimates (e.g. regression coefficient) AND variation (e.g. standard deviation) or associated estimates of uncertainty (e.g. confidence intervals)
☐	☒	For null hypothesis testing, the test statistic (e.g. *F*, *t*, *r*) with confidence intervals, effect sizes, degrees of freedom and *P* value noted<br>*Give P values as exact values whenever suitable.*
☐	☒	For Bayesian analysis, information on the choice of priors and Markov chain Monte Carlo settings
☐	☒	For hierarchical and complex designs, identification of the appropriate level for tests and full reporting of outcomes
☐	☒	Estimates of effect sizes (e.g. Cohen's *d*, Pearson's *r*), indicating how they were calculated

Our web collection on statistics for biologists contains articles on many of the points above.

Software and code

Policy information about availability of computer code

Data collection	All data were collected by UK Biobank. No specific software was written for data collection.
Data analysis	We are releasing our Machine Learning pipeline for estimating DISH from DEXA scans. In addition, we have described all analysis and modules used to analyze data within the manuscript in the Methods. In addition, we used the following softwares for data analyses:<br>BOLT-LMM version 2.3.2<br>LDSC version aa33296<br>GCTA version 1.93.2<br>twoSampleMR version 0.5.6<br>pymc3 version 3.6<br>statsmodels 0.9.0

For manuscripts utilizing custom algorithms or software that are central to the research but not yet described in published literature, software must be made available to editors and reviewers. We strongly encourage code deposition in a community repository (e.g. GitHub). See the Nature Portfolio guidelines for submitting code & software for further information.

Data

Policy information about availability of data

All manuscripts must include a data availability statement. This statement should provide the following information, where applicable:

- Accession codes, unique identifiers, or web links for publicly available datasets
- A description of any restrictions on data availability
- For clinical datasets or third party data, please ensure that the statement adheres to our policy

All raw data is distributed by the UK Biobank. The GWAS summary statistics is being released on the EBI GWAS catalog.

Human research participants

Policy information about studies involving human research participants and Sex and Gender in Research.

Reporting on sex and gender	All data in the manuscript is disaggregated at the sex level.
Population characteristics	The population varied in age between 40 and 70 years of age in the UK. Genotypic information was used in the Caucasians in the cohort. Electronic health records were collected for 3 years following baseline.
Recruitment	Participation was voluntary based on sending mail to adults in the UK and ~500,000 participants joined the cohort.
Ethics oversight	The study was approved by the UK Biobank.

Note that full information on the approval of the study protocol must also be provided in the manuscript.

Field-specific reporting

Please select the one below that is the best fit for your research. If you are not sure, read the appropriate sections before making your selection.

☒ Life sciences ☐ Behavioural & social sciences ☐ Ecological, evolutionary & environmental sciences

For a reference copy of the document with all sections, see nature.com/documents/nr-reporting-summary-flat.pdf

Life sciences study design

All studies must disclose on these points even when the disclosure is negative.

Sample size	We were able to quantify DISH in 40,346 DEXA scans collected by UK Biobank. These are based on the number of DXA scans available from imaging cohort in UK Biobank at the time the study was started.
Data exclusions	There are no data exclusions.
Replication	No replication performed in this work. There was no similar data size with genetics for DISH cohort.
Randomization	This is a general population volunteer based cohort. No further randomization was done within the cohort.
Blinding	There was no blinding as this was a study meant to understand epidemiology of different diseases and pathologies within a general population without any interventions.

Reporting for specific materials, systems and methods

We require information from authors about some types of materials, experimental systems and methods used in many studies. Here, indicate whether each material, system or method listed is relevant to your study. If you are not sure if a list item applies to your research, read the appropriate section before selecting a response.

Materials & experimental systems

n/a	Involved in the study
☒	☐ Antibodies
☒	☐ Eukaryotic cell lines
☒	☐ Palaeontology and archaeology
☒	☐ Animals and other organisms
☒	☐ Clinical data
☒	☐ Dual use research of concern

Methods

n/a	Involved in the study
☒	☐ ChIP-seq
☒	☐ Flow cytometry
☒	☐ MRI-based neuroimaging
